# mTORC2-SGK-1 acts in two environmentally responsive pathways with opposing effects on longevity

**DOI:** 10.1111/acel.12248

**Published:** 2014-07-09

**Authors:** Masaki Mizunuma, Elke Neumann-Haefelin, Natalie Moroz, Yujie Li, T Keith Blackwell

**Affiliations:** 1 Joslin Diabetes Center, Harvard Stem Cell Institute, Harvard Medical School Department of GeneticsBoston, MA, 02215, USA; 2 Department of Molecular Biotechnology, Graduate School of Advanced Sciences of Matter, Hiroshima UniversityHigashi-Hiroshima, 739-8530, Japan; 3 Renal Division, University Hospital FreiburgFreiburg, 79106, Germany; 4 Division of Biological Sciences, Department of Genetics and Complex Diseases, Harvard School of Public HealthBoston, MA, 02115, USA

**Keywords:** aging, microbiome, mTORC2, rictor, serum- and glucocorticoid-regulated kinase, SKN-1/Nrf

## Abstract

The nematode worm *Caenorhabditis elegans* provides a powerful system for elucidating how genetic, metabolic, nutritional, and environmental factors influence aging. The mechanistic target of rapamycin (mTOR) kinase is important in growth, disease, and aging and is present in the mTORC1 and mTORC2 complexes. In diverse eukaryotes, lifespan can be increased by inhibition of mTORC1, which transduces anabolic signals to stimulate protein synthesis and inhibit autophagy. Less is understood about mTORC2, which affects *C. elegans* lifespan in a complex manner that is influenced by the bacterial food source. mTORC2 regulates *C. elegans* growth, reproduction, and lipid metabolism by activating the SGK-1 kinase, but current data on SGK-1 and lifespan seem to be conflicting. Here, by analyzing the mTORC2 component Rictor (RICT-1), we show that mTORC2 modulates longevity by activating SGK-1 in two pathways that affect lifespan oppositely. RICT-1/mTORC2 limits longevity by directing SGK-1 to inhibit the stress-response transcription factor SKN-1/Nrf in the intestine. Signals produced by the bacterial food source determine how this pathway affects SKN-1 and lifespan. In addition, RICT-1/mTORC2 functions in neurons in an SGK-1-mediated pathway that increases lifespan at lower temperatures. RICT-1/mTORC2 and SGK-1 therefore oppose or accelerate aging depending upon the context in which they are active. Our findings reconcile data on SGK-1 and aging, show that the bacterial microenvironment influences SKN-1/Nrf, mTORC2 functions, and aging, and identify two longevity-related mTORC2 functions that involve SGK-regulated responses to environmental cues.

## Introduction

The mechanistic target of rapamycin (mTOR) kinase is a conserved regulator of cell growth, morphogenesis, and proliferation (Laplante & Sabatini, 2012). mTOR resides in two complexes, mTORC1 and mTORC2, which are defined by their adaptors Raptor and Rictor, respectively (Wullschleger *et al*., 2006; Laplante & Sabatini, 2012). mTORC1 senses nutrients, growth signals, and oxygen and promotes growth by increasing mRNA translation and inhibiting autophagy (Laplante & Sabatini, 2012). The biological functions of mTORC2 are poorly understood, but evidence from yeast, *Caenorhabditis elegans*, and *Drosophila* indicates that it is also important for growth (Jones *et al*., 2009; Soukas *et al*., 2009; Zinzalla *et al*., 2011; Wang *et al*., 2012). mTORC2 phosphorylates and activates the AGC-family kinases AKT, serum- and glucocorticoid-regulated kinase (SGK), and protein kinase C (PKC), which are involved in survival- and growth-related functions (Laplante & Sabatini, 2012).

mTOR is essential for development, but has been implicated in diabetes, cancer, and cardiovascular diseases, as well as aging (Wullschleger *et al*., 2006; Laplante & Sabatini, 2012; Johnson *et al*., 2013). Indeed, genetic or pharmacologic inhibition of mTOR extends lifespan in diverse model organisms (Harrison *et al*., 2009; Bjedov *et al*., 2010; Kapahi *et al*., 2010; Kenyon, 2010; Robida-Stubbs *et al*., 2012; Johnson *et al*., 2013). Lower levels of mTORC1 activity promote longevity and seem to be critical for longevity from dietary restriction (DR), and the mTORC1 inhibitor rapamycin extends lifespan in yeast, worms, flies, and mice (Harrison *et al*., 2009; Bjedov *et al*., 2010; Kapahi *et al*., 2010; Kenyon, 2010; Robida-Stubbs *et al*., 2012; Fang *et al*., 2013; Johnson *et al*., 2013). However, recent evidence indicates that rapamycin treatment also reduces mTORC2 activity *in vivo* and that some of its effects on lifespan involve mTORC2 as well as mTORC1 (Lamming *et al*., 2012; Robida-Stubbs *et al*., 2012).

Work in *C. elegans* on the Rictor ortholog *rict-1* has begun to elucidate processes that are regulated by mTORC2. *rict-1* loss-of-function mutants exhibit developmental abnormalities (slow growth, small body size, impaired reproduction, and increased fat storage), and their lifespan is markedly affected by the type of bacterial food they consume (Jones *et al*., 2009; Soukas *et al*., 2009). Individual bacterial strains vary in how well they support *C. elegans* growth and reproduction, a parameter that is not understood but has been defined as food ‘quality’ (Avery & Shtonda, 2003; Shtonda & Avery, 2006). In wild-type (WT) worms, lifespan is increased modestly by feeding of higher-quality food such as the *Escherichia coli* strain HT115 (Soukas *et al*., 2009; Maier *et al*., 2010). This difference is magnified in *rict-1* mutants, which live slightly longer than WT when fed HT115 but have a markedly reduced lifespan on the standard laboratory strain OP50 (Soukas *et al*., 2009). An understanding of this food source effect may reveal a paradigm for how an organism responds to differences in nutrient intake or to signals from the bacteria that are present in its body space (microbiome). The second possibility would be of particular interest, given that the gut microbiome profoundly influences human metabolism and immunity (Tremaroli & Bäckhed, 2012).

Loss of *rict-1* influences development as well as aging, but RICT-1/mTORC2 also modulates *C. elegans* lifespan independently of its developmental functions. When adults are exposed to *rict-1* RNA interference (RNAi) by feeding, a method that involves RNA delivery by the ‘high-quality’ strain HT115, lifespan is increased by up to 25% through activation of the transcription factor SKN-1 (Robida-Stubbs *et al*., 2012). SKN-1 is the ortholog of the mammalian Nrf1/2/3 proteins, which defend against oxidative and xenobiotic stress and have important functions in protein homeostasis (An & Blackwell, 2003; Sykiotis & Bohmann, 2010; Robida-Stubbs *et al*., 2012; Glover-Cutter *et al*., 2013). SKN-1/Nrf has been implicated in longevity in various organisms and, in *C. elegans*, is involved in lifespan extension from reduced mTORC1 or insulin/IGF-1 signaling (IIS), and DR (Tullet *et al*., 2008; Sykiotis & Bohmann, 2010; Robida-Stubbs *et al*., 2012).

Genetic evidence indicates that *rict-1* influences development, growth, and fat storage largely by activating SGK-1, the *C. elegans* ortholog of the three mammalian SGK kinases (Jones *et al*., 2009; Soukas *et al*., 2009). It is unclear whether SGK-1 might mediate RICT-1/mTORC2 effects on aging, though, given current data on how SGK-1 influences lifespan. SGK-1 inhibits SKN-1 nuclear accumulation directly, through phosphorylation, and *sgk-1* RNAi has been observed to increase lifespan dependent upon *skn-1* (Tullet *et al*., 2008). This predicts that RICT-1/mTORC2 might modulate lifespan simply by directing SGK-1 to inhibit SKN-1. However, *sgk-1* null mutants are short-lived (Soukas *et al*., 2009; Alam *et al*., 2010; Chen *et al*., 2013; Xiao *et al*., 2013), and an *sgk-1* gain-of-function (gf) mutant is long-lived (Chen *et al*., 2013). SGK-1 acts in a pathway in which a cold-sensing TRP channel (TRPA-1) and Ca^2+^ signaling increase lifespan at lower temperatures, through the action of the FoxO ortholog DAF-16 (Xiao *et al*., 2013). The role of SGK-1 in this pathway can explain results obtained with *sgk-1* mutants, but not *sgk-1* RNAi. Complicating matters further, it has been reported that longevity from *sgk-1* RNAi also requires *daf-16* (Hertweck *et al*., 2004), whereas *daf-16* is dispensable for longevity from *rict-1* RNAi or rapamycin (Robida-Stubbs *et al*., 2012). Given the apparent contradictions among these SGK-1 data, it has seemed unlikely that they can be explained within a unifying model.

To address this problem, we have investigated how *rict-1* and *sgk-1* affect SKN-1 activity, stress resistance, and lifespan under different food source and temperature conditions. Our findings reconcile the data on *sgk-1* and longevity, by showing that mTORC2 and SGK-1 act in two pathways that have opposing effects on lifespan. In the first pathway, mTORC2 directs SGK-1 to inhibit SKN-1. Depending upon cues from the bacterial microbiome, release of this inhibition may allow SKN-1 to increase stress resistance and lifespan. In the second pathway, mTORC2 activity in neurons is required for SGK-1 to promote longevity at lower temperatures. mTORC2 and SGK-1 therefore determine how mechanisms that increase lifespan are modulated by two distinct environmental inputs, bacterially produced signals and temperature.

## Results and discussion

### Food source, temperature, and SKN-1 determine how RICT-1/mTORC2 affects stress resistance and lifespan

**Figure 1:**
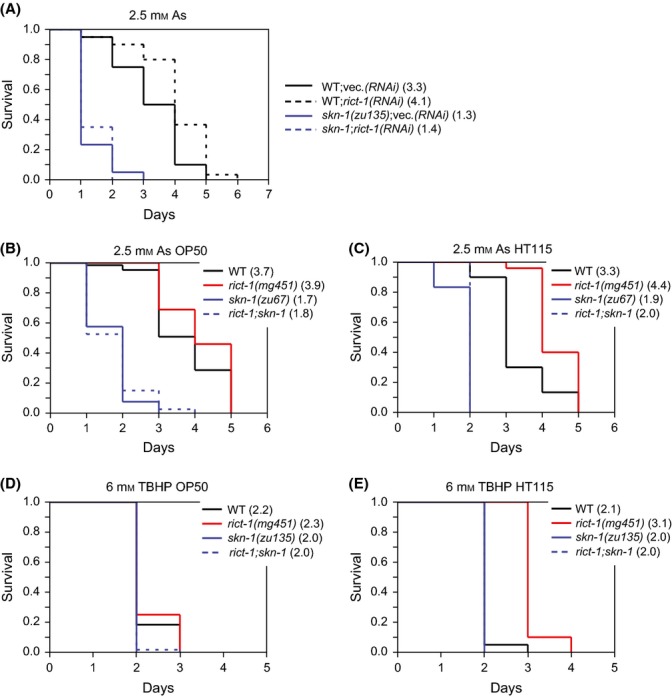
Loss of *rict-1* function increases oxidative stress resistance dependent upon food source and SKN-1. (A) *rict-1* RNAi increased resistance to oxidative stress from Arsenite (As). (B, C) Effect of *rict-1* mutation on As stress resistance, assayed in animals maintained on OP50 (B) or HT115 (C). (D, E) Effects of *rict-1* mutation on resistance to oxidative stress from tert-butyl hydrogen peroxide (TBHP), assayed as in (B, C). In these and other experiments, the *rict-1(mg451)* and *skn-1(zu135)* alleles were analyzed unless otherwise indicated. Both *rict-1(mg451)* and *skn-1(zu135)* are probable null alleles (WormBase) (Soukas *et al*., 2009). Mean survival is shown in parentheses. All stress analyses were performed at 20 °C. Representative experiments are shown, with replicates, statistics, and percent changes in survival time provided in Tables S1 (A), S2 (B, C), and S3 (D, E).

SKN-1 accumulates in intestinal nuclei in response to various stresses (An & Blackwell, 2003), but does so constitutively when *rict-1* mutants are fed the ‘quality’ strain HT115 (Robida-Stubbs *et al*., 2012). This does not occur with OP50 feeding, however, suggesting that loss of RICT-1/mTORC2 function activates SKN-1 only when animals are maintained on quality food. To test this idea further, we asked whether loss of *rict-1* increases stress resistance in a *skn-1*-dependent manner in animals that are fed HT115 and OP50 as typical examples of quality and standard food, respectively (Avery & Shtonda, 2003; Shtonda & Avery, 2006). Exposure to *rict-1* feeding RNAi (in HT115) increased resistance to oxidative stress from sodium arsenite (As) in WT animals, but not *skn-1* mutants (Fig. 1A and Table S1, Supporting information). A probable null *rict-1* mutant was also more resistant to As treatment than WT, but only when propagated on HT115 rather than OP50 (Fig. 1B,C, Table S2, Supporting information). Again, this increased stress resistance required *skn-1*. *rict-1* mutation also conferred resistance to tert-butyl hydrogen peroxide (TBHP) in a manner that was largely dependent upon *skn-1* and HT115 feeding (Fig. 1D,E, Table S3, Supporting information).

**Figure 2:**
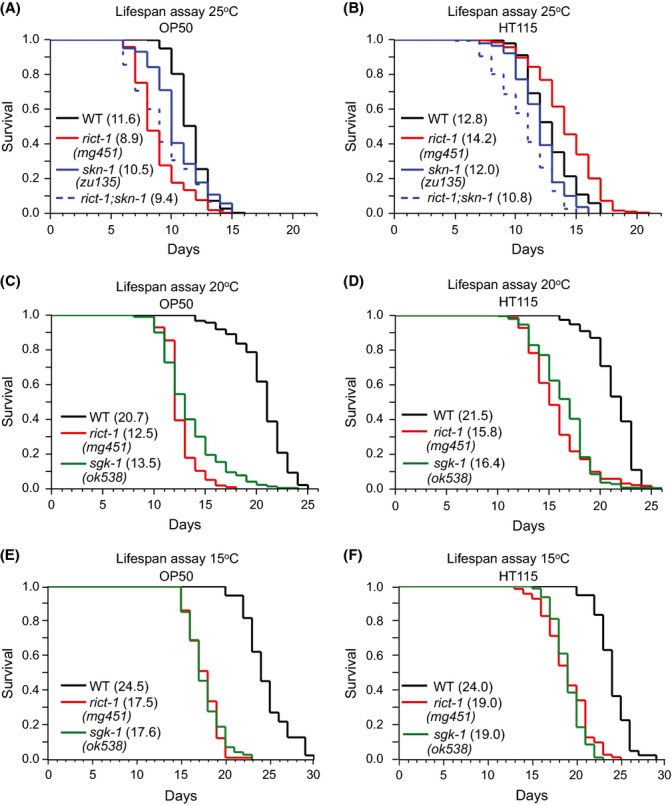
Influence of temperature and food source on *rict-1* lifespan effects. (A, B) Effects of *rict-1(mg451)* and *skn-1(zu135)* mutations on lifespan at 25 °C, in animals propagated on OP50 (A) or HT115 (B). (C–F) Lifespan analysis of *rict-1(mg451)* and *sgk-1(ok538)* mutants at 20 °C (C and D) or 15 °C (E and F), in animals propagated on OP50 (C and E) or HT115 (D and F). *sgk-1(ok538)* is a presumed null allele (Hertweck *et al*., 2004). Note that the effects of *rict-1(mg451)* and *sgk-1(ok538)* mutation were similar. Analyses of *rict-1(mg451)* and *sgk-1(ok538)* mutants performed in parallel at 25 °C are presented in Fig. 4D,E. Panels A–D each show a composite of three replicates, with mean adult lifespans indicated in parentheses. Statistics for individual and pooled experimental data are provided in Tables S4 (A, B), S5 (C, D), and S6 (E, F) (Supporting information). These lifespan experiments are compared with others performed at the same temperatures in Tables S21 and S22 (Supporting information).

We next investigated the involvement of SKN-1 in the effects of food source on *rict-1* lifespan, which had been examined only at 25 °C (Soukas *et al*., 2009). Our experiments at 25 °C reproduced these earlier findings: *rict-1* animals fed HT115 lived slightly longer than WT, but on OP50, their lifespan was significantly reduced (Fig. 2A,B, Table S4, Supporting information). By contrast, when *skn-1* was inactivated, the *rict-1* mutation failed to increase lifespan in animals that were fed HT115 (compare *rict-1; skn-1* double mutants to *skn-1* mutants, Fig. 2B and Table S4, Supporting information). Moreover, a comparison of Fig. 2A,B reveals that *rict-1* but not *rict-1; skn-1* mutants lived far longer when fed HT115 instead of OP50 (+60% vs. +15% mean lifespan). Both WT and *skn-1* mutant animals lived slightly longer on ‘quality’ food (+10% and +14%, respectively), but *skn-1* was required for the dramatically increased benefits of quality food enjoyed by *rict-1* mutants. This scenario in which quality food increases SKN-1 activity seems to be unique. For example, SKN-1 accumulation in intestinal nuclei and *skn-1*-dependent oxidative stress resistance were dramatically increased in *daf-2* (insulin/IGF-1 receptor) mutants that were propagated on OP50 (Tullet *et al*., 2008).

Given that *C. elegans* and other ectotherms live longer at lower temperature (Lee & Kenyon, 2009; Xiao *et al*., 2013), we analyzed *rict-1* lifespan at 20 °C. In contrast to the lifespan increase seen with *rict-1* RNAi (Robida-Stubbs *et al*., 2012), at 20 °C, *rict-1* mutation dramatically shortened lifespan compared with WT on either food source (Fig. 2C,D, Table S5, Supporting information). *rict-1* mutants lived longer on HT115 than OP50, but the absolute and relative differences were less pronounced at 20 °C than at 25 °C (15.8 days on HT115 vs. 12.5 days on OP50 at 20 °C (+26%), compared with 14.2 days on HT115 vs. 8.9 days on OP50 at 25 °C (+59%); Fig. 2). Similar results were obtained at 15 °C (Fig. 2E,F, Table S6, Supporting information). Thus, while WT animals lived considerably longer at lower temperatures when raised on either food, *rict-1* mutants benefitted much less from the reductions in temperature. Although reproduction is impaired in *rict-1* mutants (Jones *et al*., 2009; Soukas *et al*., 2009), the effects of *rict-1* on lifespan do not derive from a pathway in which lifespan is extended by a reduction in germline stem cell (GSC) number (Kenyon, 2010). GSC ablation extends lifespan at either 20 °C or 25 °C (Kenyon, 2010), in contrast to *rict-1* mutation (Fig. 2A–D), and *daf-16* is required for lifespan extension from GSC loss (Kenyon, 2010), but not *rict-1* RNAi (Robida-Stubbs *et al*., 2012). Moreover, *rict-1* RNAi increased lifespan in a long-lived mutant that lacks GSCs (Fig. S1, Table S18, Supporting information). Our data show that the impact of *rict-1* on longevity is profoundly influenced by temperature as well as food source and by whether *rict-1* function is impaired by mutation or RNAi.

### RICT-1/mTORC2 modulates SKN-1 activity, stress resistance, and lifespan through SGK-1

Most biological functions of *rict-1* seem to be mediated through SGK-1 (Jones *et al*., 2009; Soukas *et al*., 2009), but current data on *sgk-1* and lifespan are beset with apparent contradictions (see Introduction). Having observed that *rict-1* effects on lifespan are profoundly influenced by the bacterial food source, temperature, and the mode by which *rict-1* function is disrupted (mutation or RNAi), we incorporated these variables into an analysis of how *sgk-1* influences stress resistance and lifespan.

**Figure 3:**
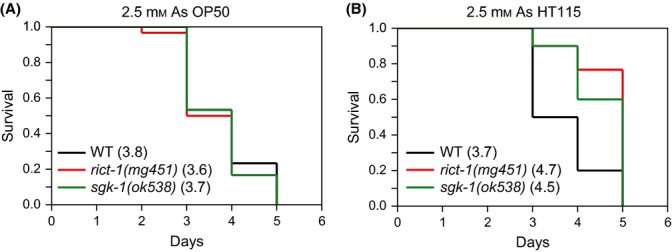
Food source dependence of *sgk-1* oxidative stress resistance. (A, B) Arsenite (As) resistance deriving from *sgk-1(ok538)* and *rict-1(mg451)* mutants in response to HT115, assayed in animals maintained on OP50 (A) or HT115 (B). Mean survival is shown in parentheses. All stress analyses were performed at 20 °C. Representative experiments are shown, with replicates, statistics, and percent changes in survival time provided in Table S7 (Supporting information).

Loss of *rict-1* or *sgk-1* affected oxidative stress resistance similarly. Knockdown of either gene increased oxidative stress resistance in a largely *skn-1*-dependent manner (Fig. 1A) (Tullet *et al*., 2008). Importantly, a *sgk-1* loss-of-function mutant was resistant to oxidative stress when maintained on HT115 but not OP50, as was true for *rict-1* mutants (Fig. 3, Table S7, Supporting information). Knockdown of either *sgk-1* or *rict-1* led to accumulation of SKN-1 in intestinal nuclei (Tullet *et al*., 2008; Robida-Stubbs *et al*., 2012) and to upregulation of well-characterized SKN-1 target genes that are involved in oxidative stress resistance (Fig. S2A–C, Supporting information). Moreover, in *sgk-1* mutants, the presence of SKN-1 in nuclei was enhanced only when they were propagated on quality food (Fig. S2D, Supporting information), analogously to the effect of *rict-1* mutation on SKN-1 nuclear accumulation (Robida-Stubbs *et al*., 2012).

**Figure 4:**
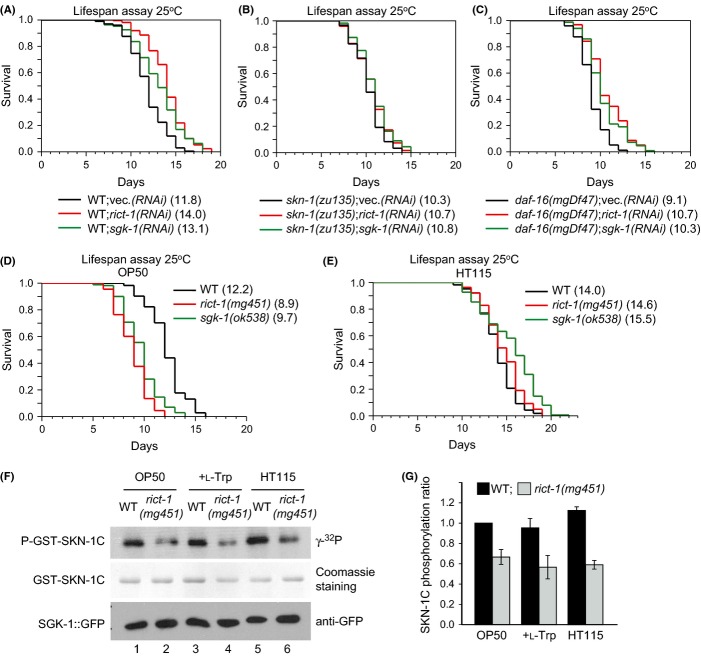
RICT-1/mTORC2 affects lifespan by acting through SGK-1. (A–C) *skn-1* but not *daf-16* is required for *rict-1* or *sgk-1* knockdown to increase lifespan. RNAi treatments were performed only during adulthood at 25 °C. Composites of three replicates are shown, with mean lifespans indicated in parentheses. (D, E) Similar effects of food source on *rict-1(mg451)* and *sgk-1(ok538)* lifespan. Statistics and individual experimental data are provided in Tables S8 (A–C) and S9 (D and E) (Supporting information). These lifespan experiments are compared with others performed at the same temperature in Table S21 (Supporting information). (F) SKN-1C is phosphorylated by SGK-1, dependent upon *rict-1*. Wild-type (WT) animals and *rict-1(mg451)* mutants were fed with OP50, HT115, or OP50 supplemented with l-Tryptophan, as indicated. SGK-1::GFP was immunoprecipitated from *Caenorhabditis elegans* lysates with anti-GFP antibodies, quantified by Western blotting and used to phosphorylate purified GST-fused SKN-1C with [γ-^32^P]ATP. (G) Quantification of the SKN-1 phosphorylation of two independent experiments (ImageJ) normalized to the levels in WT that were fed OP50. Error bars represent ±SD.

We next examined how *sgk-1* knockdown or mutation affects lifespan. Administration of *sgk-1* feeding RNAi throughout the life cycle was reported to extend lifespan, dependent upon both *daf-16* and *skn-1* (Hertweck *et al*., 2004; Tullet *et al*., 2008). Here, we performed *sgk-1* knockdown at 25 °C, the temperature used in previous studies (Hertweck *et al*., 2004; Tullet *et al*., 2008), but did so only during adulthood to exclude any developmental effects. Adulthood *sgk-1* RNAi increased lifespan modestly but reproducibly at 25 °C in WT animals and *daf-16* mutants, but not in *skn-1* mutants, analogously to *rict-1* RNAi (Fig. 4A–C, Table S8, Supporting information). We obtained comparable results when we did not include fluorodeoxyuridine (FUdR) to block reproduction (Fig. S3, Table S19, Supporting information). *rict-1* and *sgk-1* loss-of-function mutations also affected lifespan similarly. Like *rict-1* mutants, *sgk-1* mutants were short-lived on OP50 at 25 °C, but lived much longer when raised on HT115 (Fig. 4D,E, Table S9, Supporting information). As a result, under the latter conditions, *sgk-1* mutants lived longer than WT. In striking contrast, and as others have reported, we found that *sgk-1* mutants were markedly short-lived at either 15 or 20 °C on OP50 (Fig 2C,E, Tables S5 and S6, Supporting information) (Soukas *et al*., 2009; Alam *et al*., 2010; Chen *et al*., 2013; Xiao *et al*., 2013). At these lower temperatures, lifespan was only modestly increased by HT115 feeding, again consistent with results obtained with *rict-1* mutants (Fig. 2C–F).

The striking parallels between *sgk-1* and *rict-1* effects on SKN-1 activity, stress resistance, and lifespan suggest that these effects might be explained entirely through the importance of RICT-1/mTORC2 for SGK-1 activity. Supporting this idea, the extent to which SKN-1 is phosphorylated *in vitro* by *C. elegans* SGK-1 was decreased when SGK-1 was immunoprecipitated from *rict-1* mutants (Fig. 4F (lanes 1 and 2), 4G, and Fig. S4, Supporting information). We conclude that RICT-1/mTORC2 affects lifespan by activating SGK-1 and that many of its effects on lifespan involve SGK-1-mediated inhibition of SKN-1. *skn-1* was not required for developmental, growth, or reproductive phenotypes of *rict-1* mutants, however (not shown), indicating that they involve SGK-1 acting through a different mechanism.

### Parallel regulation of SKN-1 by RICT-1/SGK-1 and bacterial signals

Our results raise the question of how the effects of RICT-1/mTORC2 on SKN-1 regulation and lifespan might be influenced by the bacterial food source. *rict-1* mutants that were raised on the quality strain HB101 on plates were previously found to spend a greater percentage of time away from the bacterial lawn than WT animals (10.8% percent vs. 3.1% percent as day-one adults) (Soukas *et al*., 2009). This suggested that when *rict-1* mutants are provided with quality food, they live longer because they then consume less food and experience a DR-like state (Soukas *et al*., 2009).

**Figure 5:**
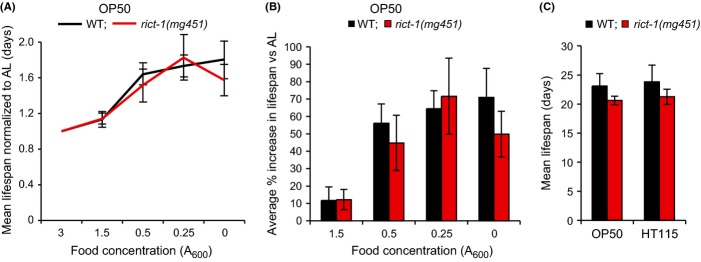
Effects of liquid culture and dietary restriction (DR) on *rict-1* mutants. (A, B) *rict-1(mg451)* mutants respond robustly to DR in a liquid bacterial dilution protocol (Experimental Procedures). *A*_600_ = 3 is designated as *ad libitum* feeding. (C) *rict-1(mg451)* lifespan is not increased by HT115 vs. OP50 feeding in liquid culture (Student’s *t*-test *P <* 0.0001). *Ad libitum* feeding at 20 °C is shown. Note that under these conditions, *rict-1(mg451)* lifespan is only modestly reduced compared with wild-type (WT). Statistical analysis is presented in Tables S10 (A, B), S11 (A, B), and S12 (C) (Supporting information).

However, we have obtained various lines of evidence suggesting that propagation on quality food may influence *rict-1* lifespan primarily through a mechanism other than DR. Under our conditions, in a microbial avoidance assay (Melo & Ruvkun, 2012), *rict-1* mutants spent a similar proportion of time away from either an HT115 or OP50 lawn (Fig. S5, Supporting information). This raised the possibility that *rict-1* mutants might consume a reduced amount of food in either case, but be unable to respond to DR when fed OP50. However, we observed that *rict-1* mutants provided with OP50 lived much longer when subjected to DR in a liquid-feeding regimen, arguing against this idea (Fig. 5A,B, Tables S10 and S11, Supporting information). Moreover, the reduction in food availability needed for robust DR lifespan extension (≥6-fold, Fig. 5A,B) was greater than would be expected to arise from the proportion of time that *rict-1* mutants spent off their food in plate culture. Unexpectedly, in liquid cultures at 20 °C, *rict-1* animals lived nearly as long as WT when fed either HT115 or OP50 *ad libitum* (Fig. 5C and Table S12, Supporting information), in striking contrast to results obtained on plates at this temperature (Fig. 2C,D). This last result suggests that when *rict-1* mutants are maintained in liquid, neither temperature nor food source influences their lifespan to the dramatic extent seen in plate culture. Together, our data suggest that the effects of quality food on SKN-1 activity and lifespan in *rict-1* mutants are less likely to derive from differences in nutrient intake than from signals produced by ‘quality’ bacteria and that these signals are perceived on plates but not in liquid.

**Figure 6:**
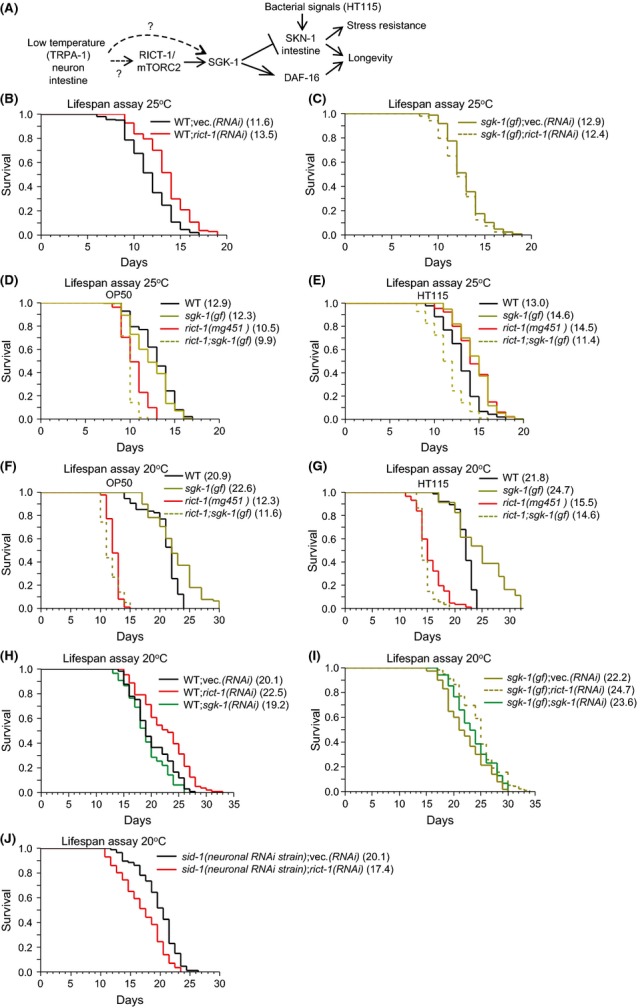
RICT-1/mTORC2 and SGK-1 act in two pathways that affect lifespan oppositely. (A) A working model for how RICT-1/mTORC2 and SGK-1 influence *Caenorhabditis elegans* longevity. RICT-1/mTORC2 directs SGK-1 to activate DAF-16 in the TRPA-1 temperature-sensing pathway and inhibit SKN-1 in an interaction that is modulated by bacterially produced signals. These signals regulate SKN-1 downstream or in parallel to its phosphorylation by SGK-1. A single interconnected pathway is arbitrarily shown, but while mTORC2 and SGK-1 inhibit SKN-1 in the intestine, neuronal mTORC2/RICT-1 is required for longevity mediated by the TRPA-1 pathway. See text for details. (B, C) The *sgk-1(gf)* mutation blocked lifespan extension from adulthood *rict-1* RNAi. (D, E) *sgk-1(gf)* dramatically reduced the lifespan extension that *rict-1* mutants experienced when fed HT115 instead of OP50. (F, G) *sgk-1(gf)* failed to increase lifespan in *rict-1(mg451)* mutants, which are short-lived at 20 °C. (H, I) Effects of *rict-1* and *sgk-1* RNAi on lifespan in *sgk-1(gf)* mutants. Compare to (G) to observe the dramatic difference between effects of *rict-1* mutation and RNAi, and note that *sgk-1* RNAi did not interfere with the lifespan increase in *sgk-1(gf)* relative to wild-type (WT) (H, I). (J) Lifespan is reduced by neuronal *rict-1* RNAi. RNAi was performed in a strain in which the dsRNAi transporter mutation *sid-1* is rescued specifically in neurons (Calixto *et al*., 2010). A composite of three replicates is shown in B–E, H, and I, with mean lifespans indicated in parentheses. Statistics and all individual experiments are described in Tables S13 (B, C), S14 (D, E), S15 (F, G), S16 (H, I), and S17 (J) (Supporting information). These lifespan experiments are compared with others performed at the same temperatures in Tables S21 and S22 (Supporting information).

Propagation on quality food was required for either *rict-1* or *sgk-1* mutation to promote SKN-1 nuclear accumulation and increase oxidative stress resistance (Fig. 1B–E, 3B, and Fig. S2D, Supporting information) (Robida-Stubbs *et al*., 2012), suggesting that signals from the bacterial food source influence SKN-1 at a step downstream of or in parallel to SGK-1 (Fig. 6A). Consistent with this notion, propagation on HT115 did not reduce SGK-1 phosphorylation of SKN-1 in either WT or *rict-1* animals (Fig. 4F (lanes 5 and 6), 4G, and Fig. S4, Supporting information). Perhaps signals produced by quality food lower a threshold for SKN-1 activation, so that when either *rict-1* or *sgk-1* function is lost under these conditions, SKN-1 accumulates in nuclei and increases stress resistance and lifespan (Fig. 6A).

Recent findings indicate that bacterial metabolites of l-Tryptophan (l-Trp) mediate effects of quality food on *C. elegans* reproduction (Gracida & Eckmann, 2013). Animals that lack NHR-114, a homolog of the hepatic transcription factor HNF4, were sterile when fed OP50 but not HT115. Remarkably, supplementation of OP50 with l-Trp rescued this phenotype. Rescue was mediated by l-Trp metabolites, which induced intestinal expression of enzymes that detoxify xenobiotics and other small molecules and are otherwise *nhr-114*-dependent. Apparently, quality food produces chemical signals that direct *C. elegans* to increase detoxification enzyme expression (Gracida & Eckmann, 2013).

SKN-1 regulates many of the same detoxification gene classes that were induced by l-Trp supplementation (Oliveira *et al*., 2009), suggesting that it might mediate this effect of l-Trp metabolites. Accordingly, l-Trp supplementation of OP50 increased SKN-1 nuclear accumulation and activity (Fig. S6A,B, Supporting information). This effect was greatly magnified in *rict-1* mutants, in which l-Trp supplementation of OP50 activated SKN-1 even more dramatically than HT115 feeding (Fig. S6C,D, Supporting information). Like HT115 feeding, l-Trp supplementation did not alter SGK-1 activity (Fig. 4F (lanes 3 and 4), 4G, and Fig. S4, Supporting information). However, l-Trp supplementation decreased the lifespan of either WT or *rict-1* animals (Fig. S7 and Table S20, Supporting information), in contrast to maintenance on HT115. Our data suggest that l-Trp metabolites induce a SKN-1-mediated detoxification response that might mediate rescue of germ cell production in OP50-fed *nhr-114* mutants, but also that l-Trp supplementation alone does not recapitulate the benefits of HT115 feeding for longevity. Perhaps quality food generates additional signals that activate SKN-1 in *rict-1* and *sgk-1* mutants and mediate its effects on lifespan, but are less toxic long term than l-Trp metabolites.

### Dual longevity functions of RICT-1/mTORC2 and SGK-1

The above models explain many of our findings, but not the profound effect of temperature on lifespan seen in *rict-1* and *sgk-1* mutants (Fig. 2). For example, on OP50 WT, animals lived almost twice as long at 20 °C compared with 25 °C (20.7 days vs. 11.5 days), but *rict-1* mutants did not benefit nearly as much from the decrease in temperature (12.5 days vs. 8.9 days)(Fig. 2A,C). Similar effects were seen in *sgk-1* mutants (Fig. 2C–F, 4D,E). The previous finding that SGK-1 is required in the TRPA-1 pathway that increases *C. elegans* lifespan at low temperature (Xiao *et al*., 2013) could explain the relative sensitivity of *sgk-1* mutants to temperatures of 20 °C or below (Fig. 6A). Our evidence that *rict-1* and *sgk-1* mutations affect lifespan and stress resistance essentially identically, and that mTORC2 promotes SGK-1 activity, predicts that RICT-1/mTORC2 also acts in the TRPA-1 pathway, by activating SGK-1 (Fig. 6A). Taken together, our findings suggest the working model that mTORC2 and SGK-1 act in two pathways that affect lifespan oppositely (Fig. 6A). They inhibit the pro-longevity and stress-response effects of SKN-1, but on the other hand promote longevity in the context of the TRPA-1 low-temperature pathway. The latter pathway appears to function independently of SKN-1 (Xiao *et al*., 2013). The balance between these two pathways would determine how mTORC2/SGK-1 influences longevity.

If the pathways suggested by our model are linear, it might be possible to compensate for lack of mTORC2 activity by activating SGK-1. To test this idea, we examined how lifespan is affected by the *sgk-1* gain-of-function allele *ft15*, which was identified in a *rict-1* suppressor screen (Jones *et al*., 2009). This *sgk-1(gf)* mutation suppressed the effects of a *rict-1* hypomorph on fat accumulation, body size, and development and partially suppressed the effects of a *rict-1* null mutation on body size, suggesting that *sgk-1(gf)* allows the SGK-1 kinase to be partially active in the absence of mTORC2 activity. Importantly, *sgk-1(gf)* prevented *rict-1* RNAi from increasing lifespan (Fig. 6B,C, Table S13, Supporting information) and substantially reduced the response of *rict-1* mutants to propagation on quality food that is seen at 25 °C (Fig. 6D,E, Table S14, Supporting information; compare *rict-1* and *rict-1; sgk-1(gf)* mutants). This strongly supports the model that RICT-1/mTORC2 inhibits SKN-1 by activating SGK-1 and that this pathway mediates the food-source-dependent effects of *rict-1* or *sgk-1* loss on lifespan and stress resistance.

Constitutive SGK-1 activation might not only compensate for the effects of reducing mTORC2 activity, but also increase lifespan through constitutive activation of the TRPA-1 temperature-sensing pathway (Fig. 6A). Consistent with this idea, at 20 °C, the *sgk-1(gf)* mutation modestly increased lifespan in a *daf-16*-dependent manner (Chen *et al*., 2013). We also found that *sgk-1(gf)* mutants lived slightly longer than WT and observed lifespan extension not only at 20 °C but also at 25 °C with HT115 feeding (Fig. 6B–G and Tables S13–S15, Supporting information). The explanation for the latter result is not clear, but it could imply that under some circumstances, the TRPA-1 pathway can increase lifespan at higher temperatures. At 25 °C with HT115 feeding, lifespan can therefore be increased modestly by either complete loss of SGK-1 (Fig. 4E) or partial SGK-1 activation (*sgk-1(gf)*; Fig. 6B,C,E). In the former case, SGK-1-mediated inhibition of intestinal SKN-1 is released, and in the latter case, any possible reduction in SKN-1 activity is presumably outweighed by benefits of increased SGK-1 function through the TRPA-1 pathway or a different mechanism. Interestingly, simultaneous loss of *rict-1* by mutation prevented *sgk-1(gf)* from increasing lifespan (Fig. 6D–G, Tables S14 and S15, Supporting information). Apparently, the partial constitutive SGK-1 activation that occurs in *sgk-1(gf)* mutants (Jones *et al*., 2009) is not sufficient to overcome the need for *rict-1* in this context.

It was striking that in plate cultures at 20 °C, lifespan was increased by *rict-1* RNAi (to 22.5d, Fig. 6H and Table S16, Supporting information) (Robida-Stubbs *et al*., 2012) but dramatically shortened by *rict-1* mutation, even with HT115 feeding (to 15.8d, Fig. 2D). In contrast to *rict-1* mutation, *rict-1* RNAi should leave RICT-1 function largely intact in neurons (Fig. 6A), because in *C. elegans*, RNAi is essentially inactive in neurons (Timmons *et al*., 2001). Given that the TRPA-1 pathway increases lifespan by acting in neurons and the intestine (Xiao *et al*., 2013), the difference in the lifespans of *rict-1(RNAi)* and *rict-1* mutant animals at 20 °C suggests that neuronal RICT-1/mTORC2 activity might be critical in the TRPA-1 pathway. Accordingly, the difference between *rict-1* mutant and RNAi lifespans was even greater in the background of the *sgk-1(gf)* mutant, in which the baseline activity of the TRPA-1 pathway should be increased: At 20 °C, *rict-1(RNAi)*; *sgk-1(gf)* animals lived 69% longer than *rict-1*; *sgk-1(gf)* double mutants that were propagated on HT115 (24.7d vs. 14.6d; Fig. 6G,I). Remarkably, the *sgk-1(gf)* mutation increased lifespan by a greater extent in *sgk-1(RNAi)* than control RNAi animals (by 4.4d vs. 2.1d; Fig. 6H,I). *sgk-1(gf)* mutants in which either *rict-1* or *sgk-1* is knocked down by RNAi therefore may benefit from both longevity pathways regulated by mTORC2/SGK-1, through increased activity of the TRPA-1 pathway in neurons, and release of mTORC2/SGK-1-mediated inhibition of SKN-1 in the intestine (Fig. 6A). Finally, *rict-1* RNAi decreased lifespan in a strain in which RNAi is active only in neurons, in striking contrast to the effect of *rict-1* RNAi on WT animals (Fig. 6H,J, and Table S17, Supporting information). We conclude that neuronal mTORC2/SGK-1 activity is critical for longevity conferred by the TRPA-1 low-temperature pathway.

### Complex homeostatic functions of mTORC2 *in vivo*

By showing that SGK-1 mediates the effects of mTORC2 on *C. elegans* stress resistance and lifespan, we have further demonstrated the importance of SGK-1 as a biological target of this little-understood but critical kinase complex. In mammals, the SGK kinases regulate numerous ion channels but may have many other overlapping functions, and have been implicated in hypertension, metabolic diseases, and cancer (Bruhn *et al*., 2013; Lang & Shumilina, 2013). Together with other *C. elegans* studies (Jones *et al*., 2009; Soukas *et al*., 2009), our new findings suggest models for how mammalian SGK kinases could be involved in mTORC2-regulated functions in growth, metabolism, stress defense, and interactions with the environment. Our data have also reconciled seemingly conflicting observations with respect to how *C. elegans* SGK-1 influences lifespan. They are consistent with all published findings on SGK-1 and longevity, except for the report that the lifespan increase from *sgk-1* RNAi was *daf-16-*dependent (Hertweck *et al*., 2004), a discrepancy that could derive from differences in RNAi conditions. Most importantly, we have shown that by activating SGK-1, mTORC2 regulates two distinct longevity pathways that have opposing effects (Fig. 6A).

In one pathway, mTORC2/SGK-1 inhibits SKN-1 (Fig. 6A). While much remains to be learned about mTORC2 functions, it is clear that this kinase complex is involved in growth (Jones *et al*., 2009; Soukas *et al*., 2009; Zinzalla *et al*., 2011; Wang *et al*., 2012). Other growth-related pathways also inhibit SKN-1 (mTORC1 and IIS) (Tullet *et al*., 2008; Robida-Stubbs *et al*., 2012), suggesting that it might be advantageous to suppress SKN-1-regulated protective mechanisms under anabolic conditions and to mobilize these stress defenses when growth signals and resources are low. IIS increases activity of the AKT kinase, which inhibits SKN-1 directly through phosphorylation (Tullet *et al*., 2008), but also activates SGK-family kinases in *C. elegans* and mammals (Hertweck *et al*., 2004; Bruhn *et al*., 2013; Lang & Shumilina, 2013). This makes it of interest to determine whether the mTORC2 and IIS pathways might converge at SGK-1 and SKN-1 under certain conditions. During the earliest embryonic stages in *C. elegans*, SKN-1 specifies endomesoderm development (Bowerman *et al*., 1992), a function that is antagonized by mTORC2/SGK-1 but not mTORC1 (Ruf *et al*., 2013), suggesting that the effects of these growth regulatory pathways on SKN-1 are both context and tissue dependent.

The striking influence of food source on *rict-1* mutant stress resistance and lifespan involves regulation of SKN-1 at a step downstream of or in parallel to SGK-1 (Fig. 6A). Apparently, signals produced by quality food lower a threshold for SKN-1 activity that determines whether reduction in mTORC2/SGK-1 signaling is sufficient to release SKN-1 from inhibition. The difference in *rict-1* lifespan that resulted from OP50 vs. HT115 feeding disappeared with liquid culture (Fig. 5C), suggesting that critical signals from these bacterial strains may be volatile, or that culture conditions influence their production or perception. Recently, neuronal signaling was implicated in a dietary adaptation to alterations in proline catabolism (Pang & Curran, 2014) and in a transcriptional response to bacterial signals that resulted in *C. elegans* developing at different rates when propagated on OP50 and the soil bacteria *Comamonas* (MacNeil *et al*., 2013). We have determined that signals from the bacterial microenvironment also modulate intestinal SKN-1 activity, particularly when mTORC2/SGK-1 activity is low. Each of these findings provides a paradigm for applying this simple model organism to investigate how interactions between a host organism and its bacterial microbiome lead to changes in gene expression, metabolism, and health. Our results also raise the exciting possibility that in mammals, the microbiome might influence the activity of Nrf proteins.

In a second longevity pathway that involves an environmental input, we found that RICT-1/mTORC2 acting in neurons is required for the TRPA-1/SGK-1 pathway to increase lifespan at low temperature (Fig. 6A). The importance of RICT-1/mTORC2 in this mechanism suggests the exciting possibility that mTORC2 might respond to either temperature or Ca^2+^ signaling (Fig. 6A). Surprisingly, under liquid culture conditions, *rict-1* mutants lived nearly as long as WT animals at 20 °C, in striking contrast to results obtained on plates (Fig. 2C,D, and 5C). Perhaps *C. elegans* adapts to lower temperatures independently of the TRPA-1 pathway in liquid culture, or a particular aspect of the liquid culture environment (reduced oxygen availability, constant thrashing) might largely circumvent the need for this pathway. Alternatively, the temperature signal itself might be influenced by immersion in liquid. Our results identify an unexpected function for RICT-1/mTORC2 in this adaptation to temperature and provide an exciting new entry point for probing the TRPA-1/SGK-1 longevity pathway and other processes that involve mTORC2. They also demonstrate how environmental inputs from culture conditions, temperature, and signals from bacteria may substantially alter how genetic pathways influence *C. elegans* aging, illustrating the value of the organism for uncovering these regulatory networks.

## Experimental procedures

Full detailed methods and experimental procedures are available in Supporting Information.

## Supporting Information

**Fig. S1** *rict-1* RNAi increases lifespan in *glp-1(bn18)* mutants.

**Fig. S2** *rict-1* or *sgk-1* knockdown activates SKN-1 target genes.

**Fig. S3** FUdR does not affect lifespan extension from *rict-1* or *sgk-1* RNAi.

**Fig. S4** Full-length gels corresponding to Fig. 4F.

**Fig. S5** *rict-1* mutants avoid OP50 and HT115 similarly.

**Fig. S6** l-Tryptophan supplementation induces SKN-1 target genes.

**Fig. S7** l-Tryptophan supplementation decreases WT lifespan.

**Table S1** Oxidative stress assays shown in Fig. 1A. **Table S2** Oxidative stress assays shown in Fig. 1B,C. **Table S3** Oxidative stress assays shown in Fig. 1D,E. **Table S4** Lifespans shown in Fig. 2A,B. **Table S5** Lifespans shown in Fig. 2C,D. **Table S6** Lifespans shown in Fig. 2E,F. **Table S7** Oxidative stress assays shown in Fig. 3 **Table S8** Lifespans shown in Fig. 4A–C. **Table S9** Lifespans shown in Fig. 4D,E. **Table S10** Lifespans shown in Fig. 5A–C **Table S11** Statistical analysis shown in Fig. 5A,B. **Table S12** Lifespans shown in Fig. 5C. **Table S13** Lifespans shown in Fig. 6B,C. **Table S14** Lifespans shown in Fig. 6D,E. **Table S15** Lifespans shown in Fig. 6F,G. **Table S16** Lifespans shown in Fig. 6H,I. **Table S17** Lifespans shown in Fig. 6J. **Table S18** Lifespans shown in Fig. S1. **Table S19** Lifespans shown in Fig. S3. **Table S20** Lifespans shown in Fig. S7. **Table S21** Summary Table of Lifespans at 25 °C **Table S22** Summary Table of Lifespans at 20 °C. **Table S23** Nematode strains used in this study.

**Appendix S1**. Experimental procedures.
