# Supplementary figures and images for: mTORC2-SGK-1 acts in two environmentally responsive pathways with opposing effects on longevity
Source: Aging Cell. 2014 Jul 9;13(5):869–78. doi: 10.1111/acel.12248 (PMC4172656; doi:10.1111/acel.12248)

Supplementary Figure 1

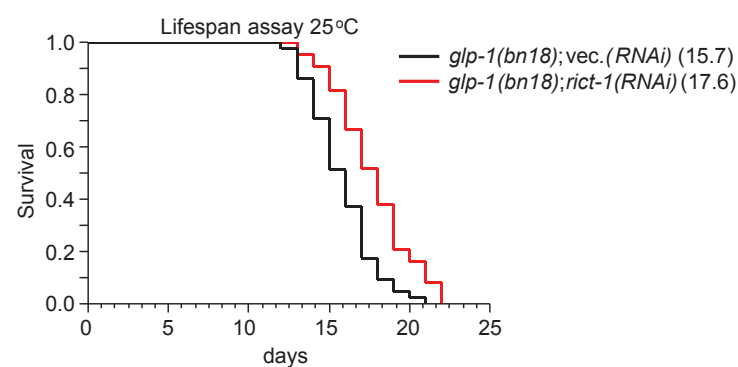

Supplement: Supplementary file 1 — Fig. S1 rict-1 RNAi increases lifespan in glp-1(bn18) mutants. [file acel0013-0869-sd1.pdf]

Supplementary Figure 2

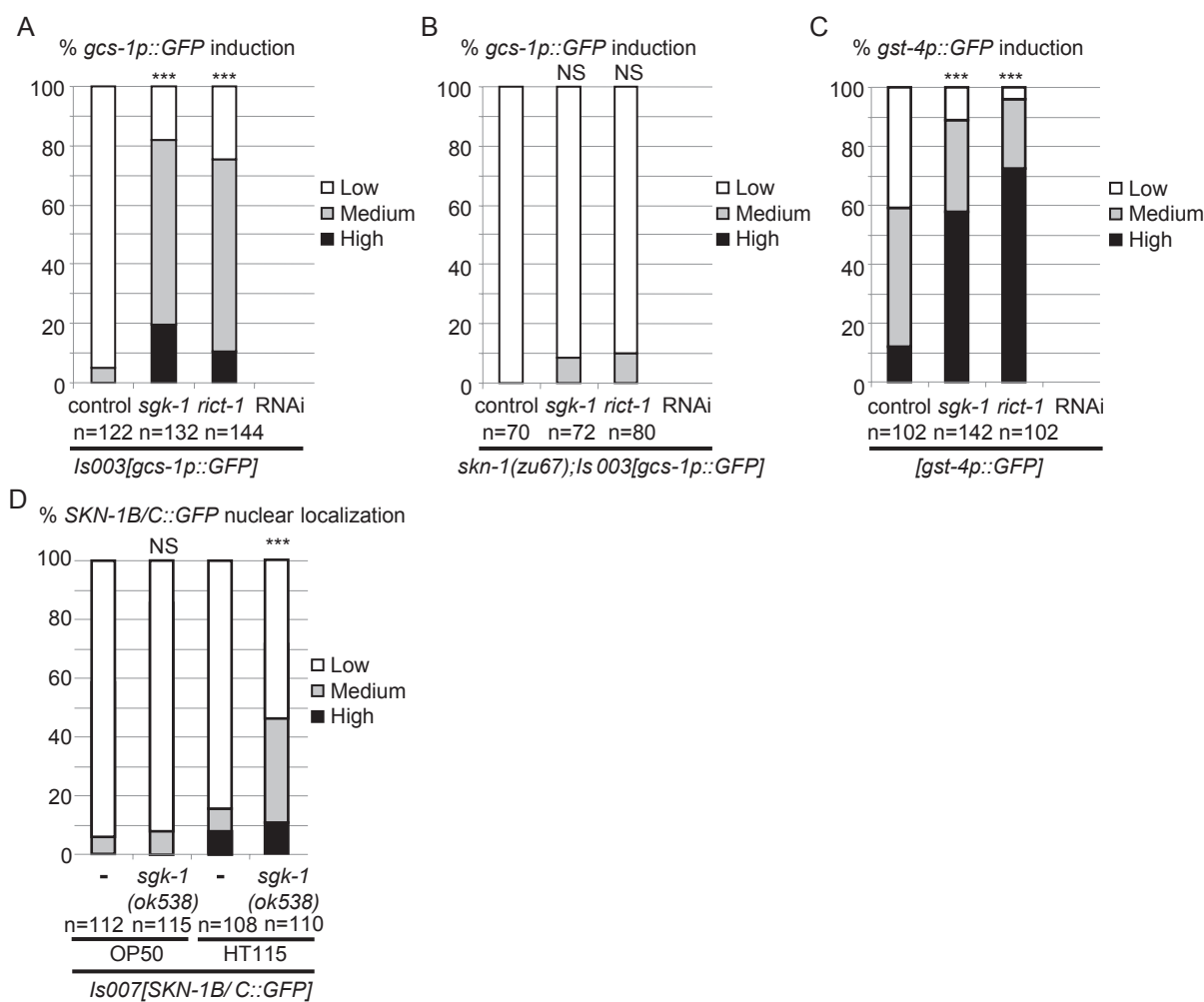

Supplement: Supplementary file 2 — Fig. S2 rict-1 or sgk-1 knockdown activates SKN-1 target genes. [file acel0013-0869-sd2.pdf]

Supplementary Figure 3

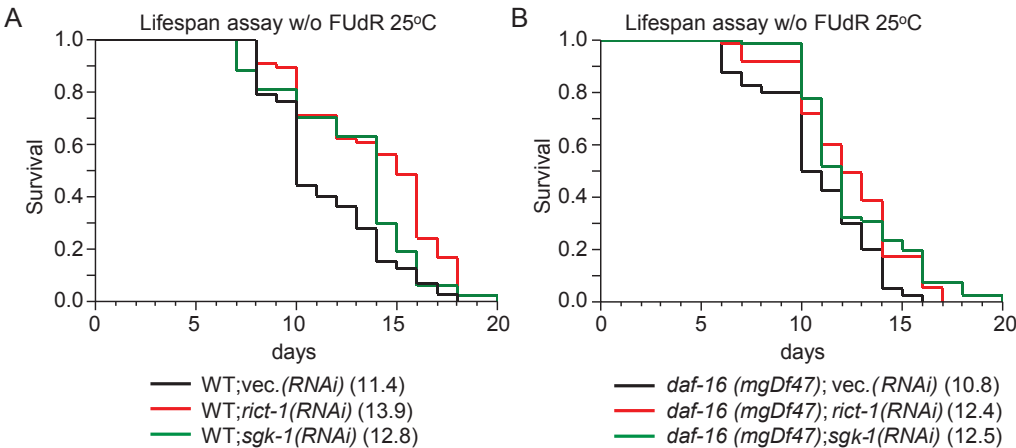

Supplement: Supplementary file 3 — Fig. S3 FUdR does not affect lifespan extension from rict-1 or sgk-1 RNAi. [file acel0013-0869-sd3.pdf]

Supplementary Figure 4

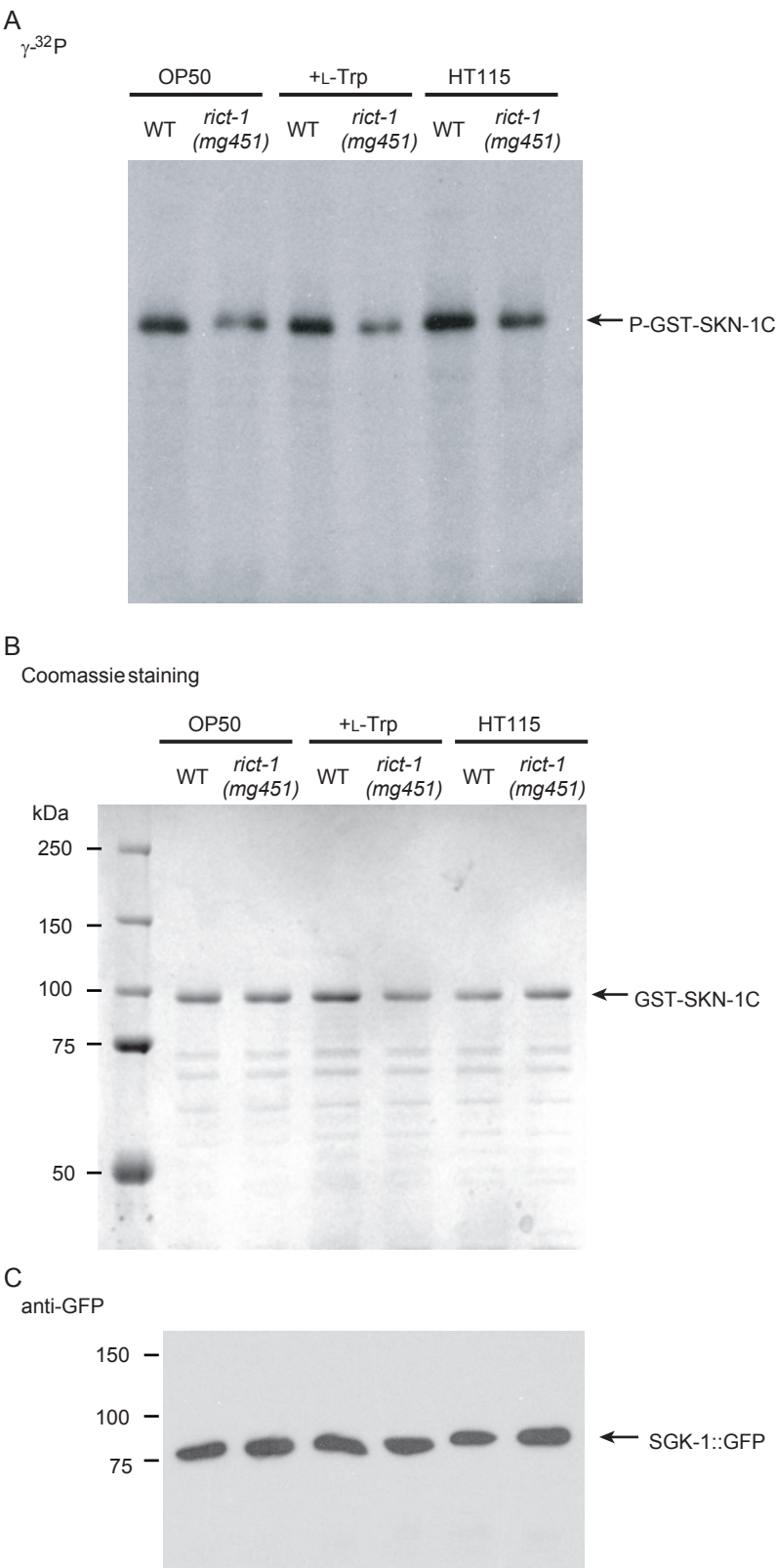

Supplement: Supplementary file 4 — Fig. S4 Full-length gels corresponding to Fig. 4F. [file acel0013-0869-sd4.pdf]

Supplementary Figure 5

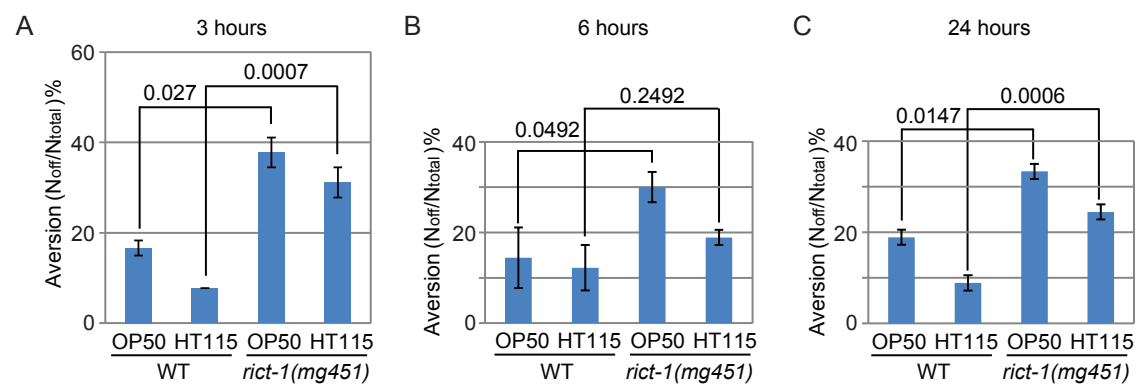

Supplement: Supplementary file 5 — Fig. S5 rict-1 mutants avoid OP50 and HT115 similarly. [file acel0013-0869-sd5.pdf]

Supplementary Figure 6

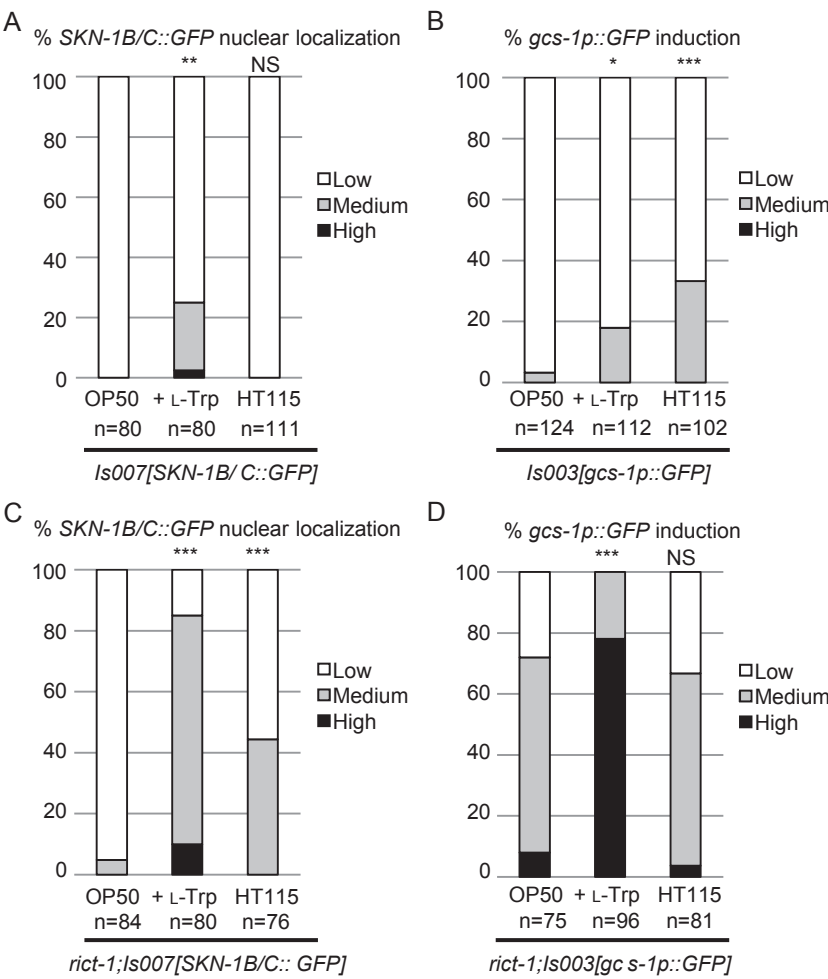

Supplement: Supplementary file 6 — Fig. S6 l-Tryptophan supplementation induces SKN-1 target genes. [file acel0013-0869-sd6.pdf]

Supplementary Figure 7

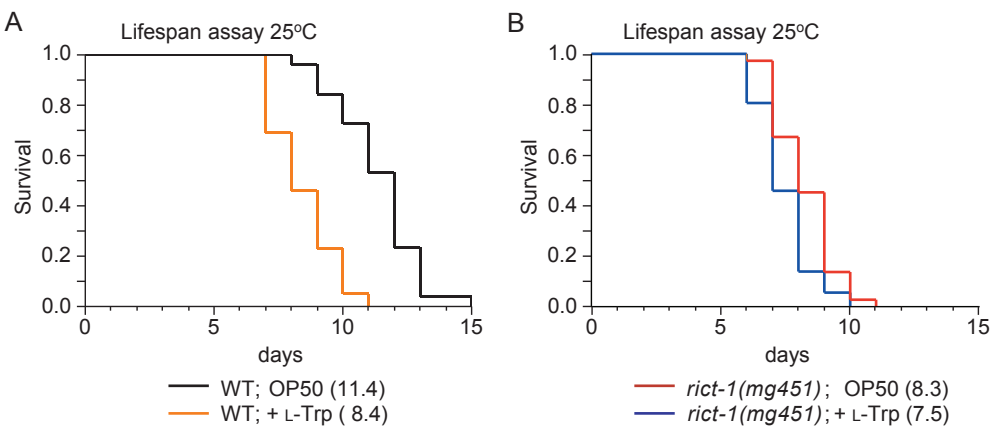

Supplement: Supplementary file 7 — Fig. S7 l-Tryptophan supplementation decreases WT lifespan. [file acel0013-0869-sd7.pdf]
