# Supplementary material for: mTORC2-SGK-1 acts in two environmentally responsive pathways with opposing effects on longevity
Source: Aging Cell. 2014 Jul 9;13(5):869–78. doi: 10.1111/acel.12248 (PMC4172656; doi:10.1111/acel.12248)
Supplement: Supplementary file 8 — Table S1 Oxidative stress assays shown in Fig. 1A. Table S2 Oxidative stress assays shown in Fig. 1B,C. Table S3 Oxidative stress assays shown in Fig. 1D,E. Table S4 Lifespans shown in Fig. 2A,B. Table S5 Lifespans shown in Fig. 2C,D. Table S6 Lifespans shown in Fig. 2E,F. Table S7 Oxidative stress assays shown in Fig. 3 Table S8 Lifespans shown in Fig. 4A–C. Table S9 Lifespans shown in Fig. 4D,E. Table S10 Lifespans shown in Fig. 5A–C Table S11 Statistical analysis shown in Fig. 5A,B. Table S12 Lifespans shown in Fig. 5C. Table S13 Lifespans shown in Fig. 6B,C. Table S14 Lifespans shown in Fig. 6D,E. Table S15 Lifespans shown in Fig. 6F,G. Table S16 Lifespans shown in Fig. 6H,I. Table S17 Lifespans shown in Fig. 6J. Table S18 Lifespans shown in Fig. S1. Table S19 Lifespans shown in Fig. S3. Table S20 Lifespans shown in Fig. S7. Table S21 Summary Table of Lifespans at 25 °C Table S22 Summary Table of Lifespans at 20 °C. Table S23 Nematode strains used in this study. [file acel0013-0869-sd8.docx]

**Table S1. Oxidative stress assays shown in Figure 1A**

**Arsenite (As) stress**

| Strain | RNAi treatment | RNAi mean survival (days±SEM) | 75^th^  percentile  (days) | No. RNAi animals | % Mean survival extension | *P* value against control | Assay  # | Figure |
| --- | --- | --- | --- | --- | --- | --- | --- | --- |
| WT | Control | 3.30±0.14 | 4 | 60/60 | N/A | N/A | 1 | 1A |
|  | *rict-1* | 4.05±0.14 | 5 | 60/60 | 23* | < .0001* | 1 | 1A |
| *skn-1(zu135)* | Control | 1.28±0.07 | 1 | 40/40 | N/A | N/A | 1 | 1A |
|  | *rict-1* | 1.35±0.06 | 2 | 40/40 | 5** | .5184** | 1 | 1A |
| WT | Control | 3.30±0.14 | 4 | 60/60 | N/A | N/A | 2 |  |
|  | *rict-1* | 4.05±0.14 | 5 | 60/60 | 23* | < .0001* | 2 |  |
| *skn-1(zu135)* | Control | 1.20±0.05 | 1 | 40/40 | N/A | N/A | 2 |  |
|  | *rict-1* | 1.32±0.06 | 2 | 40/40 | 10** | .1460** | 2 |  |
| WT | Control | 3.25±0.15 | 4 | 60/60 | N/A | N/A | 3 |  |
|  | *rict-1* | 4.20±0.15 | 5 | 60/60 | 29* | < .0001* | 3 |  |
| *skn-1(zu135)* | Control | 1.28±0.08 | 1 | 40/40 | N/A | N/A | 3 |  |
|  | *rict-1* | 1.32±0.06 | 2 | 40/40 | 3** | .9403** | 3 |  |

(*) WT control vs. *rict-1(RNAi)*, (**) *skn-1* control vs. *skn-1*;*rict-1(RNAi)*

Assay numbers indicate trials that were performed in parallel. pL4440 empty vector plates were used for RNAi controls. L4 stage animals were placed on RNAi plates and allowed to lay eggs. Young F1 adults were transferred to M9 containing 2.5 mM As, then periodically scored for survival. *P* values were obtained by log-rank.

**Table S2. Oxidative stress assays shown in Figures 1B and 1C**

**Arsenite (As) stress**

| E. coli | Strain | Mean survival (days±SEM) | 75^th^  percentile  (days) | No. animals | % Mean survival extension | *P* value against control | Assay  # | Figure |
| --- | --- | --- | --- | --- | --- | --- | --- | --- |
| OP50 bacteria | WT | 3.73±0.12 | 5 | 63/63 | N/A | N/A | 1 | 1B |
|  | *rict-1(mg451)* | 4.15±0.11 | 5 | 61/61 | 11* | .0215* | 1 | 1B |
|  | *skn-1(zu67)* | 1.65±0.10 | 2 | 40/40 | N/A | N/A | 1 | 1B |
|  | *rict-1(mg451);*  *skn-1(zu67)* | 1.76±0.13 | 2 | 41/41 | 7** | .4779** | 1 | 1B |
|  | WT | 3.30±0.13 | 4 | 60/60 | N/A | N/A | 2 |  |
|  | *rict-1(mg451)* | 3.93±0.10 | 5 | 60/60 | 19* | .0018* | 2 |  |
|  | *skn-1(zu67)* | 1.52±0.07 | 2 | 58/58 | N/A | N/A | 2 |  |
|  | *rict-1(mg451);*  *skn-1(zu67)* | 1.57±0.06 | 2 | 60/60 | 3** | .5916** | 2 |  |
|  | WT | 3.57±0.23 | 5 | 30/30 | N/A | N/A | 3 |  |
|  | *rict-1(mg451)* | 3.87±0.17 | 5 | 30/30 | 8* | .5389* | 3 |  |
|  | *skn-1(zu67)* | 1.67±0.10 | 2 | 39/40 | N/A | N/A | 3 |  |
|  | *rict-1(mg451);*  *skn-1(zu67)* | 1.93±0.15 | 2 | 40/40 | 16** | .1418** | 3 |  |
| HT115 bacteria | WT | 3.33±0.11 | 4 | 60/60 | N/A | N/A | 1 | 1C |
|  | *rict-1(mg451)* | 4.36±0.08 | 5 | 50/50 | 31* | < .0001* | 1 | 1C |
|  | *skn-1(zu67)* | 1.83±0.05 | 2 | 60/60 | N/A | N/A | 1 | 1C |
|  | *rict-1(mg451);*  *skn-1(zu67)* | 2.00 | 2 | 40/40 | 9** | .0068** | 1 | 1C |
|  | WT | 3.70±0.13 | 4 | 61/61 | N/A | N/A | 2 |  |
|  | *rict-1(mg451)* | 4.52±0.10 | 5 | 60/60 | 22* | < .0001* | 2 |  |
|  | *skn-1(zu67)* | 1.37±0.07 | 2 | 40/40 | N/A | N/A | 2 |  |
|  | *rict-1(mg451);*  *skn-1(zu67)* | 1.60±0.08 | 2 | 54/55 | 17** | .0282** | 2 |  |
|  | WT | 3.49±0.11 | 4 | 59/60 | N/A | N/A | 3 |  |
|  | *rict-1(mg451)* | 4.23±0.09 | 5 | 60/60 | 21* | < .0001* | 3 |  |
|  | *skn-1(zu67)* | 1.77±0.08 | 2 | 31/40 | N/A | N/A | 3 |  |
|  | *rict-1(mg451);*  *skn-1(zu67)* | 1.83±0.06 | 2 | 36/40 | 3** | .5447** | 3 |  |

(*) WT vs. *rict-1*, (**) *skn-1* vs. *rict-1;skn-1*

Assay numbers indicate trials that were performed in parallel. Animals were fed with OP50 or HT115 for two generations, then young F1 adults were transferred to M9 solution that contained 2.5 mM As and periodically scored for survival. All data were analyzed using JMP software. *P* values were obtained by log-rank.

**Table S3. Oxidative stress assays shown in Figures 1D and 1E**

**t-butyl hydrogen peroxide (TBHP) stress**

| E. coli | Strain | Mean survival (days±SEM) | 75^th^  percentile  (days) | No. animals | % Mean survival extension | *P* value against control | Assay  # | Figure |
| --- | --- | --- | --- | --- | --- | --- | --- | --- |
| OP50 bacteria | WT | 2.18±0.05 | 2 | 60/60 | N/A | N/A | 1 | 1D |
|  | *rict-1(mg451)* | 2.25±0.06 | 2.5 | 60/60 | 3* | .3774* | 1 | 1D |
|  | *skn-1(zu135)* | 2.00 | 2 | 40/40 | N/A | N/A | 1 | 1D |
|  | *rict-1(mg451);*  *skn-1(zu135)* | 2.02±0.02 | 2 | 38/40 | 1** | .3173** | 1 | 1D |
|  | WT | 2.10±0.04 | 2 | 60/60 | N/A | N/A | 2 |  |
|  | *rict-1(mg451)* | 2.22±0.05 | 2 | 60/60 | 6* | .0813* | 2 |  |
|  | *skn-1(zu135)* | 2.00 | 2 | 40/40 | N/A | N/A | 2 |  |
|  | *rict-1(mg451);*  *skn-1(zu135)* | 2.05±0.03 | 2 | 37/40 | 3** | .0807** | 2 |  |
|  | WT | 2.15±0.05 | 2 | 60/60 | N/A | N/A | 3 |  |
|  | *rict-1(mg451)* | 2.23±0.06 | 2 | 60/60 | 4* | .2482* | 3 |  |
|  | *skn-1(zu135)* | 1.98±0.02 | 2 | 40/40 | N/A | N/A | 3 |  |
|  | *rict-1(mg451);*  *skn-1(zu135)* | 2.03±0.02 | 2 | 37/40 | 3** | .0841** | 3 |  |
| HT115 bacteria | WT | 2.05±0.03 | 2 | 60/60 | N/A | N/A | 1 | 1E |
|  | *rict-1( mg451)* | 3.10±0.04 | 3 | 60/60 | 51* | < .0001* | 1 | 1E |
|  | *skn-1(zu135)* | 2.00 | 2 | 40/40 | N/A | N/A | 1 | 1E |
|  | *rict-1(mg451);*  *skn-1(zu135)* | 2.00 | 2 | 40/40 | 0** |  | 1 | 1E |
|  | WT | 2.13±0.04 | 2 | 60/60 | N/A | N/A | 2 |  |
|  | *rict-1(mg451)* | 3.07±0.03 | 3 | 60/60 | 44* | < .0001* | 2 |  |
|  | *skn-1(zu135)* | 2.00 | 2 | 40/40 | N/A | N/A | 2 |  |
|  | *rict-1(mg451);*  *skn-1(zu135)* | 2.00 | 2 | 40/40 | 0** |  | 2 |  |
|  | WT | 2.07±0.03 | 2 | 60/60 | N/A | N/A | 3 |  |
|  | *rict-1(mg451)* | 3.12±0.05 | 3 | 60/60 | 51* | < .0001* | 3 |  |
|  | *skn-1(zu135)* | 2.00 | 2 | 40/40 | N/A | N/A | 3 |  |
|  | *rict-1(mg451);*  *skn-1(zu135)* | 2.02±0.01 | 2 | 36/40 | 1** | .1556** | 3 |  |

(*) WT vs. *rict-1*, (**) *skn-1* vs. *rict-1;skn-1*

Assay numbers indicate trials that were performed in parallel. Animals were fed with OP50 or HT115 for two generations, then young F1 adults were transferred to M9 solution that contained 6 mM TBHP and periodically scored for survival. *P* values were obtained by log-rank.

**Table S4. Lifespans shown in Figures 2A and 2B**

**25°C**

| E. coli | Strain | Mean lifespan (days±SEM) | 75^th^  percentile  (days) | No. animals | % Mean lifespan extension | *P* value against control | No. of Exp. | Figure |
| --- | --- | --- | --- | --- | --- | --- | --- | --- |
| OP50 bacteria | WT | 11.61±0.07 | 13 | 334/340 | N/A | N/A | 3 | 2A |
|  | *rict-1(mg451)* | 8.87±0.11 | 10 | 330/336 | -24* | < .0001* | 3 | 2A |
|  | *skn-1(zu135)* | 10.46±0.17 | 12 | 158/170 | N/A | N/A | 3 | 2A |
|  | *rict-1(mg451);*  *skn-1(zu135)* | 9.38±0.19 | 12 | 180/190 | -10** | < .0009** | 3 | 2A |
| HT115  bacteria | WT | 12.83±0.11 | 14 | 324/330 | N/A | N/A | 3 | 2B |
|  | *rict-1(mg451)* | 14.15±0.14 | 16 | 319/330 | 10* | < .0001* | 3 | 2B |
|  | *skn-1(zu135)* | 11.97±0.16 | 13 | 140/140 | N/A | N/A | 3 | 2B |
|  | *rict-1(mg451);*  *skn-1(zu135)* | 10.78±0.18 | 13 | 156/162 | -10** | < .0001** | 3 | 2B |

(*) WT vs. *rict-1*, (**) *skn-1* vs. *rict-1;skn-1*

Data shown are a composite of 3 individual experiments, which are described below. Animals from the parental generation were grown on OP50 or HT115. Synchronized young F1 adults were transferred to lifespan plates containing 5-fluoro-2′-deoxyuridine (FUdR). *P* values were obtained by log-rank.

| E. coli | Strain | Mean lifespan (days±SEM) | 75^th^  percentile  (days) | No. animals | % Mean lifespan extension | *P* value against control | Assay  # |
| --- | --- | --- | --- | --- | --- | --- | --- |
| OP50 bacteria | WT | 12.39±0.14 | 13 | 88/90 | N/A | N/A | 1 |
|  | *rict-1(mg451)* | 9.76±0.21 | 11 | 96/96 | -21* | < .0001* | 1 |
|  | *skn-1(zu135)* | 10.57±0.31 | 12 | 49/60 | N/A | N/A | 1 |
|  | *rict-1(mg451);*  *skn-1(zu135)* | 9.23±0.41 | 11.5 | 52/60 | -13** | .2033** | 1 |
|  | WT | 11.03±0.11 | 12 | 118/120 | N/A | N/A | 2 |
|  | *rict-1(mg451)* | 8.36±0.14 | 10 | 117/120 | -24* | < .0001* | 2 |
|  | *skn-1(zu135)* | 9.96±0.31 | 9 | 49/50 | N/A | N/A | 2 |
|  | *rict-1(mg451);*  *skn-1(zu135)* | 8.87±0.26 | 10 | 60/60 | -11** | .0051** | 2 |
|  | WT | 11.60±0.11 | 12 | 128/130 | N/A | N/A | 3 |
|  | *rict-1(mg451)* | 8.63±0.17 | 10 | 117/120 | -26* | < .0001* | 3 |
|  | *skn-1(zu135)* | 10.78±0.27 | 12 | 60/60 | N/A | N/A | 3 |
|  | *rict-1(mg451);*  *skn-1(zu135)* | 9.96±0.31 | 12 | 68/70 | -8** | .0081** | 3 |
| HT115 bacteria | WT | 14.48±0.16 | 16 | 90/90 | N/A | N/A | 1 |
|  | *rict-1(mg451)* | 15.29±0.28 | 17 | 85/90 | 6* | < .0001* | 1 |
|  | *skn-1(zu135)* | 12.90±0.25 | 14 | 40/40 | N/A | < .0001 | 1 |
|  | *rict-1(mg451);*  *skn-1(zu135)* | 10.89±0.44 | 13 | 35/40 | -25** | < .0001** | 1 |
|  | WT | 11.73±0.12 | 13 | 116/120 | N/A | N/A | 2 |
|  | *rict-1(mg451)* | 12.65±0.17 | 14 | 118/120 | 8* | < .0001* | 2 |
|  | *skn-1(zu135)* | 10.58±0.33 | 11 | 40/40 | N/A | .0007 | 2 |
|  | *rict-1(mg451);*  *skn-1(zu135)* | 10.58±0.29 | 12 | 62/62 | 0** | .7253** | 2 |
|  | WT | 12.66±0.19 | 14 | 118/120 | N/A | N/A | 3 |
|  | *rict-1(mg451)* | 14.83±0.22 | 17 | 116/120 | 17* | < .0001* | 3 |
|  | *skn-1(zu135)* | 12.28±0.18 | 13 | 60/60 | N/A | N/A | 3 |
|  | *rict-1(mg451);*  *skn-1(zu135)* | 10.93±0.28 | 13 | 59/60 | -11** | .0022** | 3 |

Assay numbers indicate trials that were performed in parallel.

**Table S5. Lifespans shown in Figures 2C and 2D**

**20°C**

| E. coli | Strain | Mean lifespan (days±SEM) | 75^th^  percentile  (days) | No. animals | % Mean lifespan extension | *P* value against WT | No. of Exp. | Figure |
| --- | --- | --- | --- | --- | --- | --- | --- | --- |
| OP50 bacteria | WT | 20.68±0.18 | 22 | 183/270 | N/A | N/A | 3 | 2C |
|  | *rict-1(mg451)* | 12.52±0.10 | 13 | 213/270 | -40 | < .0001 | 3 | 2C |
|  | *sgk-1(ok538)* | 13.46±0.17 | 15 | 220/270 | -35 | < .0001 | 3 | 2C |
| HT115  bacteria | WT | 21.45±0.16 | 23 | 154/270 | N/A | N/A | 3 | 2D |
|  | *rict-1(mg451)* | 15.78±0.19 | 17 | 221/270 | -26 | < .0001 | 3 | 2D |
|  | *sgk-1(ok538)* | 16.43±0.16 | 18 | 245/270 | -23 | < .0001 | 3 | 2D |

Data shown are a composite of 3 individual experiments, which are described below. Animals from the parental generation were grown on OP50 or HT115. Synchronized young F1 adults were transferred to lifespan plates containing 5-fluoro-2′-deoxyuridine (FUdR). *P* values were obtained by log-rank.

| E. coli | Strain | Mean lifespan (days±SEM) | 75^th^  percentile  (days) | No. animals | % Mean lifespan extension | *P* value against WT | Assay  # |
| --- | --- | --- | --- | --- | --- | --- | --- |
| OP50 bacteria | WT | 20.52±0.34 | 22 | 42/90 | N/A | N/A | 1 |
|  | *rict-1(mg451)* | 11.55±0.20 | 12 | 55/90 | -44 | < .0001 | 1 |
|  | *sgk-1(ok538)* | 12.42±0.26 | 14 | 84/90 | -40 | < .0001 | 1 |
|  | WT | 20.63±0.30 | 22 | 65/90 | N/A | N/A | 2 |
|  | *rict-1(mg451)* | 13.36±0.18 | 14 | 81/90 | -35 | < .0001 | 2 |
|  | *sgk-1(ok538)* | 12.54±0.24 | 13 | 67/90 | -39 | < .0001 | 2 |
|  | WT | 20.82±0.29 | 22 | 76/90 | N/A | N/A | 3 |
|  | *rict-1(mg451)* | 12.34±0.080 | 13 | 77/90 | -35 | < .0001 | 3 |
|  | *sgk-1(ok538)* | 15.64±0.35 | 17 | 69/90 | -25 | < .0001 | 3 |
| HT115 bacteria | WT | 21.28±0.23 | 22 | 54/90 | N/A | N/A | 1 |
|  | *rict-1(mg451)* | 14.85±0.29 | 16 | 89/90 | -30 | < .0001 | 1 |
|  | *sgk-1(ok538)* | 16.00±0.29 | 18 | 84/90 | -25 | < .0001 | 1 |
|  | WT | 21.49±0.25 | 23 | 59/90 | N/A | N/A | 2 |
|  | *rict-1(mg451)* | 17.39±0.32 | 19 | 82/90 | -19 | < .0001 | 2 |
|  | *sgk-1(ok538)* | 16.58±0.31 | 18 | 79/90 | -23 | < .0001 | 2 |
|  | WT | 21.61±0.36 | 23 | 41/90 | N/A | N/A | 3 |
|  | *rict-1(mg451)* | 14.80±0.24 | 16 | 50/90 | -32 | < .0001 | 3 |
|  | *sgk-1(ok538)* | 16.72±0.25 | 18 | 82/90 | -23 | < .0001 | 3 |

Assay numbers indicate trials that were performed in parallel.

**Table S6. Lifespans shown in Figures 2E and 2F**

**15°C**

| E. coli | Strain | Mean lifespan (days±SEM) | 75^th^  percentile  (days) | No. animals | % Mean lifespan extension | *P* value against WT | No. of Exp. | Figure |
| --- | --- | --- | --- | --- | --- | --- | --- | --- |
| OP50 bacteria | WT | 24.57±0.28 | 26 | 77/90 | N/A | N/A | 1 | 2E |
|  | *rict-1(mg451)* | 17.56±0.19 | 19 | 80/90 | -29 | < .0001 | 1 | 2E |
|  | *sgk-1(ok538)* | 17.62±0.24 | 19 | 68/90 | -28 | < .0001 | 1 | 2E |
| HT115  bacteria | WT | 24.06±0.20 | 25 | 79/90 | N/A | N/A | 1 | 2F |
|  | *rict-1(mg451)* | 19.00±0.17 | 21 | 70/90 | -21 | < .0001 | 1 | 2F |
|  | *sgk-1(ok538)* | 19.03±0.19 | 20 | 80/90 | -21 | < .0001 | 1 | 2F |

Animals from the parental generation were grown on OP50 or HT115. Synchronized young F1 adults were transferred to lifespan plates containing 5-fluoro-2′-deoxyuridine (FUdR). *P* values were obtained by log-rank.

**Table S7. Oxidative stress assays shown in Figure 3**

**Arsenite (As) stress**

| E. coli | Strain | Mean survival (days±SEM) | 75^th^  percentile  (days) | No. animals | % Mean survival extension | *P* value against WT | Assay  # | Figure |
| --- | --- | --- | --- | --- | --- | --- | --- | --- |
| OP50 bacteria | WT | 3.77±0.15 | 4 | 30/30 | N/A | N/A | 1 | 3A |
|  | *rict-1(mg451)* | 3.63±0.15 | 4 | 30/30 | -4 | .5247 | 1 | 3A |
|  | *sgk-1(ok538)* | 3.70±0.14 | 4 | 30/30 | -2 | .6762 | 1 | 3A |
|  | WT | 3.73±0.13 | 4 | 30/30 | N/A | N/A | 2 |  |
|  | *rict-1(mg451)* | 3.90±0.16 | 5 | 30/30 | 5 | .2784 | 2 |  |
|  | *sgk-1(ok538)* | 3.78±0.10 | 4 | 30/30 | 1 | .5271 | 2 |  |
|  | WT | 3.77±0.14 | 4 | 30/30 | N/A | N/A | 3 |  |
|  | *rict-1(mg451)* | 3.97±0.16 | 5 | 30/30 | 5 | .2796 | 3 |  |
|  | *sgk-1(ok538)* | 3.90±0.15 | 5 | 30/30 | 3 | .4627 | 3 |  |
| HT115 bacteria | WT | 3.70±0.15 | 4 | 30/30 | N/A | N/A | 1 | 3B |
|  | *rict-1(mg451)* | 4.70±0.12 | 5 | 30/30 | 27 | < .0001 | 1 | 3B |
|  | *sgk-1(ok538)* | 4.50±0.12 | 5 | 30/30 | 22 | .0003 | 1 | 3B |
|  | WT | 3.70±0.15 | 4 | 30/30 | N/A | N/A | 2 |  |
|  | *rict-1(mg451)* | 4.70±0.12 | 5 | 30/30 | 27 | < .0001 | 2 |  |
|  | *sgk-1(ok538)* | 4.40±0.14 | 5 | 30/30 | 19 | .0005 | 2 |  |
|  | WT | 3.63±0.13 | 4 | 30/30 | N/A | N/A | 3 |  |
|  | *rict-1(mg451)* | 4.60±0.12 | 5 | 30/30 | 27 | < .0001 | 3 |  |
|  | *sgk-1(ok538)* | 4.33±0.14 | 5 | 30/30 | 19 | .0005 | 3 |  |

Assay numbers indicate trials that were performed in parallel. Animals were fed with OP50 or HT115 for two generations, then young F1 adults were transferred to M9 solution that contained 2.5 mM As and periodically scored for survival. *P* values were obtained by log-rank.

**Table S8. Lifespans shown in Figures 4A-C**

**25°C**

| Strain | RNAi treatment | RNAi mean lifespan (days±SEM) | 75^th^  percentile  (days) | No. RNAi animals | % Mean lifespan extension | *P* value against control | No. of Exp. | Figure |
| --- | --- | --- | --- | --- | --- | --- | --- | --- |
| WT | Control | 11.83±0.15 | 13 | 201/210 | N/A | N/A | 3 | 4A |
|  | *rict-1* | 14.02±0.15 | 15 | 210/212 | 19* | < .0001* | 3 | 4A |
|  | *sgk-1* | 13.12±0.21 | 15 | 189/210 | 11* | < .0001* | 3 | 4A |
| *skn-1(zu135)* | Control | 10.28±0.15 | 11 | 118/130 | N/A | N/A | 3 | 4B |
|  | *rict-1* | 10.65±0.17 | 12 | 124/131 | 4** | .0314** | 3 | 4B |
|  | *sgk-1* | 10.84±0.18 | 12 | 111/120 | 5** | .0066** | 3 | 4B |
| *daf-16 (mgDf47)* | Control | 9.06±0.09 | 10 | 250/270 | N/A | N/A | 3 | 4C |
|  | *rict-1* | 10.66±0.13 | 12 | 254/270 | 18*** | < .0001*** | 3 | 4C |
|  | *sgk-1* | 10.29±0.13 | 11 | 236/270 | 14*** | < .0001*** | 3 | 4C |

(*) WT control vs. *rict-1(RNAi)* or *sgk-1(RNAi)*, (**) *skn-1* control vs. *skn-1*;*rict-1(RNAi)* or *skn-1*;*sgk-1(RNAi)*, (***) *daf-16* control vs. *daf-16*;*rict-1(RNAi)* or *daf-16*;*sgk-1(RNAi)*

A composite of 3 individual experiments is tabulated, with individual trials described below. Animals from the parental generation were grown on HT115. RNAi treatments were performed during only adulthood, with pL4440 empty vector plates used for RNAi controls. *P* values were obtained by log-rank.

| Strain | RNAi treatment | RNAi mean lifespan (days±SEM) | 75^th^  percentile  (days) | No. RNAi animals | % Mean lifespan extension | *P* value against control | Assay  # |
| --- | --- | --- | --- | --- | --- | --- | --- |
| WT | Control | 12.35±0.19 | 14 | 89/90 | N/A | N/A | 1 |
|  | *rict-1* | 14.28±0.26 | 16 | 92/92 | 16* | < .0001* | 1 |
|  | *sgk-1* | 13.86±0.21 | 15 | 73/90 | 12* | < .0001* | 1 |
| *skn-1(zu135)* | Control | 11.08±0.28 | 13 | 36/40 | N/A | N/A | 1 |
|  | *rict-1* | 11.56±0.29 | 13 | 41/41 | 4** | .1504** | 1 |
|  | *sgk-1* | 11.86±0.29 | 12 | 36/40 | 7** | .0407** | 1 |
| *daf-16 (mgDf47)* | Control | 10.28±0.14 | 11 | 82/90 | N/A | N/A | 1 |
|  | *rict-1* | 12.84±0.15 | 14 | 82/90 | 25*** | < .0001*** | 1 |
|  | *sgk-1* | 12.27±0.19 | 13 | 79/90 | 19*** | < .0001*** | 1 |
| WT | Control | 11.64±0.29 | 13 | 55/60 | N/A | N/A | 2 |
|  | *rict-1* | 13.67±0.28 | 15 | 60/60 | 17* | < .0001* | 2 |
|  | *sgk-1* | 12.70±0.38 | 14.5 | 60/60 | 9* | .0059* | 2 |
| *skn-1(zu135)* | Control | 9.83±0.25 | 11 | 42/50 | N/A | N/A | 2 |
|  | *rict-1* | 10.22±0.26 | 11 | 46/50 | 4** | .2442** | 2 |
|  | *sgk-1* | 10.30±0.29 | 11 | 37/40 | 5** | .2231** | 2 |
| *daf-16 (mgDf47)* | Control | 8.42±0.12 | 9 | 85/90 | N/A | N/A | 2 |
|  | *rict-1* | 9.39±0.15 | 10 | 84/90 | 12*** | < .0001*** | 2 |
|  | *sgk-1* | 9.15±0.17 | 10 | 76/90 | 9*** | < .0001*** | 2 |
| WT | Control | 11.21±0.31 | 12 | 57/60 | N/A | N/A | 3 |
|  | *rict-1* | 13.80±0.30 | 15 | 58/60 | 23* | < .0001* | 3 |
|  | *sgk-1* | 12.61±0.42 | 14 | 56/60 | 12* | .0038* | 3 |
| *skn-1(zu135)* | Control | 9.88±0.17 | 11 | 40/40 | N/A | N/A | 3 |
|  | *rict-1* | 10.30±0.32 | 11 | 37/40 | 4** | .0769** | 3 |
|  | *sgk-1* | 10.39±0.28 | 11 | 38/40 | 5** | .0700** | 3 |
| *daf-16 (mgDf47)* | Control | 8.49±0.12 | 9 | 83/90 | N/A | N/A | 3 |
|  | *rict-1* | 9.83±0.17 | 10 | 88/90 | 16*** | < .0001*** | 3 |
|  | *sgk-1* | 9.44±0.14 | 10 | 81/90 | 11*** | < .0001*** | 3 |

Assay numbers indicate trials that were performed in parallel.

**Table S9. Lifespans shown in Figures 4D and 4E**

**25°C**

| E. coli | Strain | Mean lifespan (days±SEM) | 75^th^  percentile  (days) | No. animals | % Mean lifespan extension | *P* value against WT | No. of Exp. | Figure |
| --- | --- | --- | --- | --- | --- | --- | --- | --- |
| OP50 bacteria | WT | 12.19±0.14 | 13 | 175/180 | N/A | N/A | 3 | 4D |
|  | *rict-1(mg451)* | 8.88±0.14 | 10 | 178/180 | -27 | < .0001 | 3 | 4D |
|  | *sgk-1(ok538)* | 9.66±0.14 | 11 | 171/180 | -18 | < .0001 | 3 | 4D |
| HT115  bacteria | WT | 13.99±0.15 | 15 | 165/180 | N/A | N/A | 3 | 4E |
|  | *rict-1(mg451)* | 14.64±0.17 | 16 | 163/180 | 5 | < .0001 | 3 | 4E |
|  | *sgk-1(ok538)* | 15.45±0.23 | 18 | 177/180 | 10 | < .0001 | 3 | 4E |

A composite of 3 individual experiments is tabulated, with individual trials described below. Animals from the parental generation were grown on OP50 or HT115. Synchronized young F1 adults were transferred to lifespan plates. *P* values were obtained by log-rank.

| E. coli | Strain | Mean  lifespan (days±SEM) | 75^th^  percentile  (days) | No. animals | % Mean lifespan extension | *P* value against WT | Assay  # |
| --- | --- | --- | --- | --- | --- | --- | --- |
| OP50 bacteria | WT | 11.95±0.25 | 13 | 57/60 | N/A | N/A | 1 |
|  | *rict-1(mg451)* | 7.93±0.15 | 9 | 59/60 | -34 | < .0001 | 1 |
|  | *sgk-1(ok538)* | 9.67±0.26 | 11 | 60/60 | -19 | < .0001 | 1 |
|  | WT | 12.17±0.26 | 13 | 59/60 | N/A | N/A | 2 |
|  | *rict-1(mg451)* | 9.20±0.22 | 10 | 60/60 | -24 | < .0001 | 2 |
|  | *sgk-1(ok538)* | 9.53±0.24 | 11 | 57/60 | -22 | < .0001 | 2 |
|  | WT | 12.44±0.20 | 13 | 59/60 | N/A | N/A | 3 |
|  | *rict-1(mg451)* | 9.49±0.15 | 10 | 59/60 | -24 | < .0001 | 3 |
|  | *sgk-1(ok538)* | 9.80±0.21 | 11 | 54/60 | -21 | < .0001 | 3 |
| HT115 bacteria | WT | 13.67±0.23 | 15 | 57/60 | N/A | N/A | 1 |
|  | *rict-1(mg451)* | 14.32±0.28 | 16 | 57/60 | 5 | .00197 | 1 |
|  | *sgk-1(ok538)* | 15.58±0.44 | 18 | 60/60 | 14 | < .0001 | 1 |
|  | WT | 13.27±0.26 | 14 | 52/60 | N/A | N/A | 2 |
|  | *rict-1(mg451)* | 13.78±0.24 | 15 | 54/60 | 4 | .1887 | 2 |
|  | *sgk-1(ok538)* | 15.00±0.37 | 17 | 59/60 | 13 | < .0001 | 2 |
|  | WT | 15.00±0.26 | 16 | 56/60 | N/A | N/A | 3 |
|  | *rict-1(mg451)* | 14.80±0.24 | 17.5 | 52/60 | -1 | .0060 | 3 |
|  | *sgk-1(ok538)* | 15.80±0.38 | 18 | 58/60 | 5 | .0025 | 3 |

Assay numbers indicate trials that were performed in parallel.

**Table S10. Lifespans shown in Figures 5A and 5B**

| Strain | OD  600 | Mean lifespan (days) | 75^th^  Percentile  (days) | No. animals | % increase lifespan vs. AL | Mean WT lifespan (days) | 75^th^  percentile  (days) | No. WT animals | WT % change in lifespan DR vs. AL | *rict-1* % change in lifespan vs. WT | Normalized *rict-1* Mean Lifespan (days) | Normalized WT Mean Lifespan (days) | ID |
| --- | --- | --- | --- | --- | --- | --- | --- | --- | --- | --- | --- | --- | --- |
| *rict-1 (mg451)* | 3.00 | 23.30 | 26 | 40 | 0.00 | 21.87 | 26 | 31 | 0.00 | 6.53 | 1.00 | 1.00 | 12a |
|  | 0.50 | 25.69 | 28 | 36 | 10.26 | 33.09 | 41 | 34 | 51.30 | -22.36 | 1.10 | 1.51 | 12a |
|  | 0.25 | 27.80 | 33 | 51 | 19.31 | 37.70 | 47 | 30 | 72.37 | -26.26 | 1.19 | 1.72 | 12a |
|  | 0.05 | 28.68 | 33 | 34 | 23.09 | 40.06 | 47 | 34 | 83.16 | -28.41 | 1.23 | 1.83 | 12a |
|  | 0.00 | 25.44 | 29 | 32 | 9.18 | 40.35 | 47 | 37 | 84.49 | -36.95 | 1.09 | 1.84 | 12a |
| *rict-1 (mg451)* | 3.00 | 19.80 | 22 | 35 | 0.00 | 22.91 | 28 | 32 | 0.00 | -13.58 | 1.00 | 1.00 | 12b |
|  | 1.50 | 24.41 | 26 | 32 | 23.26 | 23.28 | 30 | 36 | 1.60 | 4.85 | 1.23 | 1.02 | 12b |
|  | 0.50 | 34.09 | 35 | 56 | 72.17 | 36.43 | 43 | 35 | 58.99 | -6.42 | 1.72 | 1.59 | 12b |
|  | 0.25 | 44.38 | 54 | 42 | 124.15 | 40.93 | 50 | 37 | 78.66 | 8.42 | 2.24 | 1.79 | 12b |
|  | 0.00 | 36.21 | 46 | 33 | 82.89 | 49.13 | 57 | 31 | 114.42 | -26.29 | 1.83 | 2.14 | 12b |
| *rict-1 (mg451)* | 3.00 | 19.52 | 22 | 31 | 0.00 | 22.91 | 28 | 32 | 0.00 | -14.82 | 1.00 | 1.00 | 12c |
|  | 1.50 | 22.98 | 24 | 45 | 17.74 | 23.28 | 30 | 36 | 1.60 | -1.29 | 1.18 | 1.02 | 12c |
|  | 0.50 | 37.89 | 48 | 37 | 94.16 | 36.43 | 43 | 35 | 58.99 | 4.02 | 1.94 | 1.59 | 12c |
|  | 0.25 | 46.07 | 53 | 40 | 136.07 | 40.93 | 50 | 37 | 78.66 | 12.55 | 2.36 | 1.79 | 12c |
|  | 0.00 | 39.88 | 48 | 58 | 104.34 | 49.13 | 57 | 31 | 114.42 | -18.83 | 2.04 | 2.14 | 12c |
| *rict-1 (mg451)* | 3.00 | 18.75 | 22 | 32 | 0.00 | 19.57 | 22 | 35 | 0.00 | -4.20 | 1.00 | 1.00 | 12d |
|  | 1.50 | 22.15 | 29 | 34 | 18.12 | 26.29 | 30 | 34 | 34.35 | -15.77 | 1.18 | 1.34 | 12d |
|  | 0.50 | 31.07 | 35 | 45 | 65.69 | 40.79 | 46 | 28 | 108.39 | -23.83 | 1.66 | 2.08 | 12d |
|  | 0.25 | 35.68 | 39 | 38 | 90.32 | 39.48 | 45 | 33 | 101.75 | -9.63 | 1.90 | 2.02 | 12d |
|  | 0.00 | 30.94 | 35 | 34 | 65.02 | 42.08 | 50 | 39 | 114.99 | -26.47 | 1.65 | 2.15 | 12d |
| *rict-1 (mg451)* | 3.00 | 20.11 | 23 | 28 | 0.00 | 24.85 | 28 | 25 | 0.00 | -19.09 | 1.00 | 1.00 | 12e |
|  | 1.50 | 19.55 | 21 | 29 | -2.40 | 28.84 | 35 | 35 | 14.32 | -32.21 | 0.97 | 1.16 | 12e |
|  | 0.50 | 23.34 | 26 | 29 | 14.01 | 35.25 | 43 | 44 | 37.33 | -33.77 | 1.16 | 1.42 | 12e |
|  | 0.25 | 28.79 | 33 | 29 | 37.59 | 33.54 | 38 | 35 | 31.20 | -14.16 | 1.43 | 1.35 | 12e |
|  | 0.00 | 29.13 | 35 | 30 | 39.06 | 31.72 | 38 | 36 | 24.66 | -8.16 | 1.45 | 1.28 | 12e |
|  | 0.00 | 27.80 | 35 | 30 | 33.29 | 31.72 | 38 | 36 | 24.66 | -12.36 | 1.38 | 1.28 | 12e |

Analyses of individual DR experiments performed on *rict-1* animals. Experiments with different ID numbers were performed independently of one another, sometimes weeks or months apart. Normalized value = mean lifespan at a given OD600 / mean lifespan at OD600 3.00, for a particular strain within that experiment.

**Table S11. Statistical analysis of compiled data of *rict-1* animals on DR shown in Figures 5A and 5B**

*rict-1* Mean lifespan (days)

| OD600 | Average | SEM | *t*-test vs. AL | *t*-test vs. WT |
| --- | --- | --- | --- | --- |
| 3.00 | 20.29 | 0.88 | N/A | 0.1049 |
| 1.50 | 22.27 | 1.18 | 0.1614 | 0.1108 |
| 0.50 | 30.42 | 2.98 | 0.0066 | 0.0769 |
| 0.25 | 36.55 | 4.25 | 0.0031 | 0.6389 |
| 0.00 | 31.57 | 2.44 | 0.0017 | 0.0410 |

*rict-1* 75^th^percentile (days)

| OD600 | Average | SEM | *t*-test vs. AL | *t*-test vs. WT |
| --- | --- | --- | --- | --- |
| 3.00 | 22.90 | 0.87 | N/A | 0.0373 |
| 1.50 | 25.00 | 1.94 | 0.2625 | 0.0246 |
| 0.50 | 34.40 | 4.31 | 0.0192 | 0.0558 |
| 0.25 | 42.40 | 5.22 | 0.0033 | 0.2634 |
| 0.00 | 37.92 | 3.35 | 0.0019 | 0.0587 |

*rict-1* % increase lifespan vs AL

| OD600 | Average | SEM | *t*-test vs. AL | *t-test* vs. WT |
| --- | --- | --- | --- | --- |
| 3.00 | 0.00 | 0.00 | N/A | N/A |
| 1.50 | 12.20 | 5.76 | 0.0272 | 0.9574 |
| 0.50 | 44.80 | 15.89 | 0.0135 | 0.5300 |
| 0.25 | 71.66 | 21.83 | 0.0063 | 0.7467 |
| 0.00 | 49.83 | 13.12 | 0.0045 | 0.3011 |

WT Mean lifespan (days)

| OD600 | Average | SEM | *t*-test vs. AL |
| --- | --- | --- | --- |
| 3.00 | 22.42 | 0.96 | N/A |
| 1.50 | 25.42 | 1.55 | 0.0910 |
| 0.50 | 36.40 | 1.40 | 0.0000 |
| 0.25 | 38.52 | 1.54 | 0.0000 |
| 0.00 | 40.69 | 3.49 | 0.0007 |

WT 75^th^ percentile (days)

| OD600 | Average | SEM | *t*-test vs. AL |
| --- | --- | --- | --- |
| 3.00 | 26.40 | 1.30 | N/A |
| 1.50 | 31.25 | 1.44 | 0.0257 |
| 0.50 | 43.20 | 0.89 | 0.0000 |
| 0.25 | 46.00 | 2.47 | 0.0001 |
| 0.00 | 47.83 | 3.83 | 0.0005 |

WT % increase lifespan vs AL

| OD600 | Average | SEM | *t*-test vs. AL |
| --- | --- | --- | --- |
| 3.00 | 0.00 | 0.00 | N/A |
| 1.50 | 11.73 | 7.79 | 0.0881 |
| 0.50 | 56.15 | 11.01 | 0.0005 |
| 0.25 | 64.43 | 10.38 | 0.0001 |
| 0.00 | 70.95 | 16.67 | 0.0023 |

Normalized *rict-1* Mean Lifespan (days)

| OD600 | Average | SEM | *t*-test vs. AL | *t*-test vs. WT |
| --- | --- | --- | --- | --- |
| 3.00 | 1.00 | 0.00 | N/A | 0.05 |
| 1.50 | 1.14 | 0.07 | 0.03 | 0.95 |
| 0.50 | 1.52 | 0.18 | 0.01 | 0.56 |
| 0.25 | 1.83 | 0.25 | 0.01 | 0.72 |
| 0.00 | 1.57 | 0.17 | 0.00 | 0.32 |

Normalized WT Mean Lifespan (days)

| OD600 | Average | SEM | *t*-test vs. AL |
| --- | --- | --- | --- |
| 3.00 | 1.00 | 0.00 | N/A |
| 1.50 | 1.13 | 0.09 | 0.09 |
| 0.50 | 1.64 | 0.13 | 0.00 |
| 0.25 | 1.73 | 0.12 | 0.00 |
| 0.00 | 1.81 | 0.21 | 0.00 |

*rict-1* vs. WT, % increase lifespan

| OD600 | Average | SEM | *t*-test vs. AL |
| --- | --- | --- | --- |
| 3.00 | -8.01 | 4.55 | N/A |
| 1.50 | -10.04 | 8.61 | 0.8070 |
| 0.50 | -15.21 | 6.96 | 0.3613 |
| 0.25 | -5.35 | 7.48 | 0.7427 |
| 0.00 | -20.06 | 4.43 | 0.0670 |

This table shows a composite of all individual experiments in Table S10, and corresponds to the data in Figure 5Aand 5B. The joint impact of genotype and food concentration on lifespan was assessed with two-way ANOVA tests performed in Stata, using mean and standard error values obtained from the Kaplan–Meier survival curves.

**Table S12. Lifespans shown in Figure 5C**

| Strain/  Condition | Mean lifespan (days) | Avg. | SEM | 75^th^  percentile  (days) | No. animals | % increase lifespan vs. OP50 | *t*-test vs. OP50 | % increase lifespan vs. WT | *t*-test vs. WT | ID | Figure |
| --- | --- | --- | --- | --- | --- | --- | --- | --- | --- | --- | --- |
| WT/OP50 | 19.57 | 23.09 | 2.16 | 22 | 35 |  |  |  |  | 12f | 5C |
| WT/OP50 | 24.85 |  |  | 28 | 25 |  |  |  |  | 12g | 5C |
| WT/OP50 | 24.85 |  |  | 30 | 36 |  |  |  |  | 12h | 5C |
| WT/ HT115 | 19.31 | 23.83 | 2.88 | 22 | 42 | -1.16 | 0.8152 |  |  | 12f | 5C |
| WT/ HT115 | 27.21 |  |  | 28 | 29 | 8.45 |  |  |  | 12g | 5C |
| WT/ HT115 | 24.96 |  |  | 30 | 31 | 0.38 |  |  |  | 12h | 5C |
| *rict-1(mg451)*/OP50 | 20.11 | 20.63 | 0.73 | 23 | 28 |  |  | -17.04 | 0.36 | 12g | 5C |
| *rict-1(mg451)*/OP50 | 21.15 |  |  | 23 | 32 |  |  | -13.32 |  | 12h | 5C |
| *rict-1(mg451)*/HT115 | 19.10 | 21.25 | 1.32 | 19 | 21 | -4.38 | 0.6957 | -26.85 | 0.38 | 12g | 5C |
| *rict-1(mg451)*/HT115 | 22.22 |  |  | 27 | 26 | 4.46 |  | -9.80 |  | 12h-1 | 5C |
| *rict-1(mg451)*/HT115 | 22.43 |  |  | 25 | 28 | 5.31 |  | -9.06 |  | 12h-2 | 5C |

Analyses of individual *rict-1* AL OP50 and HT115 liquid culture lifespan experiments. Experiments with different ID numbers were performed independently of each other. The 75^th^ percentile refers to the day at which 75% of the population was dead. Percentage increase in lifespan = (mean lifespan at given OD600 - mean lifespan at OD600 3.00)/mean lifespan at OD600 3.00. Number of animals represents number of observed deaths. SEM represents standard error of the mean = standard deviation/square root (N-1), when N is the number of animals tested. P values were calculated by log-rank.

**Table S13. Lifespans shown in Figures 6B and 6C**

**25°C**

| Strain | RNAi treatment | RNAi mean lifespan (days±SEM) | 75^th^  percentile  (days) | No. RNAi animals | % Mean lifespan extension | *P* value against control | No. of Exp. | Figure |
| --- | --- | --- | --- | --- | --- | --- | --- | --- |
| WT | Control | 11.63±0.15 | 13 | 243/270 | N/A | N/A | 3 | 6B |
|  | *rict-1* | 13.45±0.16 | 15 | 244/270 | 16* | < .0001* | 3 | 6B |
| *sgk-1(gf)* | Control | 12.91±0.12 | 14 | 244/270 | N/A | N/A | 3 | 6C |
|  | *rict-1* | 12.40±0.13 | 14 | 241/270 | -4** | .0232** | 3 | 6C |

(*) WT control vs. *rict-1(RNAi)*, (**) *sgk-1* control vs. *sgk-1*;*rict-1(RNAi)*.

A composite of 3 individual experiments is tabulated, with individual trials described below. Animals from the parental generation were grown on HT115. Synchronized young F1 adults were transferred to lifespan plates. RNAi treatments were performed during only adulthood, with pL4440 empty vector plates used for RNAi controls. *P* values were obtained by log-rank.

| Strain | RNAi treatment | RNAi mean lifespan (days±SEM) | 75^th^  percentile  (days) | No. RNAi animals | % Mean lifespan extension | *P* value against control | Assay  # |
| --- | --- | --- | --- | --- | --- | --- | --- |
| WT | Control | 11.91±0.28 | 14 | 74/90 | N/A | N/A | 1 |
|  | *rict-1* | 13.90±0.28 | 17 | 82/90 | 17* | < .0001* | 1 |
| *sgk-1(gf)* | Control | 13.14±0.25 | 15 | 84/90 | N/A | N/A | 1 |
|  | *rict-1* | 12.23±0.28 | 14 | 77/90 | -7** | .049** | 1 |
| WT | Control | 11.23±0.30 | 14 | 79/90 | N/A | N/A | 2 |
|  | *rict-1* | 13.69±0.28 | 15 | 74/90 | 22* | < .0001* | 2 |
| *sgk-1(gf)* | Control | 13.34±0.23 | 14 | 79/90 | N/A | N/A | 2 |
|  | *rict-1* | 12.75±0.24 | 14 | 83/90 | -4** | .1457** | 2 |
| WT | Control | 11.75±0.21 | 13 | 90/90 | N/A | N/A | 3 |
|  | *rict-1* | 12.85±0.25 | 14 | 88/90 | 9* | .0002* | 3 |
| *sgk-1(gf)* | Control | 12.25±0.21 | 13 | 85/90 | N/A | N/A | 3 |
|  | *rict-1* | 12.20±0.15 | 13 | 89/90 | -0.4** | .8555** | 3 |

Assay numbers indicate trials that were performed in parallel.

**Table S14. Lifespans shown in Figures 6D and 6E**

**25°C**

| E. coli | Strain | Mean lifespan (days±SEM) | 75^th^  percentile  (days) | No. animals | % Mean lifespan extension | *P* value against control | No. of Exp. | Figure |
| --- | --- | --- | --- | --- | --- | --- | --- | --- |
| OP50 bacteria | WT | 12.91±0.13 | 14 | 239/270 | N/A | N/A | 3 | 6D |
|  | *sgk-1(gf)* | 12.30±0.14 | 14 | 225/270 | -5* | .0065* | 3 | 6D |
|  | *rict-1(mg451)* | 10.49±0.08 | 11 | 266/270 | N/A | N/A | 3 | 6D |
|  | *rict-1(mg451);*  *sgk-1(gf)* | 9.86±0.05 | 10 | 232/270 | -6** | < .0001** | 3 | 6D |
| HT115  bacteria | WT | 13.02±0.12 | 14 | 255/270 | N/A | N/A | 3 | 6E |
|  | *sgk-1(gf)* | 14.58±0.12 | 16 | 250/270 | 12* | < .0001* | 3 | 6E |
|  | *rict-1(mg451)* | 14.45±0.15 | 16 | 195/210 | N/A | N/A | 3 | 6E |
|  | *rict-1(mg451);*  *sgk-1(gf)* | 11.44±0.13 | 13 | 196/210 | -21** | < .0001** | 3 | 6E |

(*) WT vs. *sgk-1*, (**) *rict-1* vs. *rict-1;sgk-1*

A composite of 3 individual experiments is tabulated, with individual trials described below. Animals from the parental generation were grown on OP50 or HT115. Synchronized young F1 adults were transferred to lifespan plates. *P* values were obtained by the log-rank.

| E. coli | Strain | Mean lifespan (days±SEM) | 75^th^  percentile  (days) | No. animals | % Mean lifespan extension | *P* value against control | Assay  # |
| --- | --- | --- | --- | --- | --- | --- | --- |
| OP50 bacteria | WT | 13.03±0.23 | 14.5 | 80/90 | N/A | N/A | 1 |
|  | *sgk-1(gf)* | 12.18±0.28 | 14 | 71/90 | -7* | .0504* | 1 |
|  | *rict-1(mg451)* | 10.33±0.14 | 11 | 89/90 | N/A | N/A | 1 |
|  | *rict-1(mg451);*  *sgk-1(gf)* | 9.73±0.44 | 10 | 73/90 | -6** | .0002** | 1 |
|  | WT | 13.29±0.23 | 15 | 77/90 | N/A | N/A | 2 |
|  | *sgk-1(gf)* | 12.78±0.22 | 14 | 72/90 | -4* | .0304* | 2 |
|  | *rict-1(mg451)* | 10.79±0.13 | 11 | 88/90 | N/A | N/A | 2 |
|  | *rict-1(mg451);*  *sgk-1(gf)* | 9.88±0.07 | 10 | 83/90 | -8** | < .0001** | 2 |
|  | WT | 12.44±0.23 | 14 | 82/90 | N/A | N/A | 3 |
|  | *sgk-1(gf)* | 11.99±0.24 | 14 | 82/90 | -4* | .05692* | 3 |
|  | *rict-1(mg451)* | 10.40±0.15 | 11 | 89/90 | N/A | N/A | 3 |
|  | *rict-1(mg451);*  *sgk-1(gf)* | 9.97±0.07 | 10 | 76/90 | -4** | .0031** | 3 |
| HT115 bacteria | WT | 13.29±0.26 | 15 | 82/90 | N/A | N/A | 1 |
|  | *sgk-1(gf)* | 14.38±0.23 | 16 | 84/90 | 8* | .0049* | 1 |
|  | *rict-1(mg451)* | 13.91±0.24 | 16 | 79/90 | N/A | N/A | 1 |
|  | *rict-1(mg451); sgk-1(gf)* | 10.91±0.26 | 12 | 79/90 | -22** | < .0001** | 1 |
|  | WT | 12.93±0.20 | 14 | 88/90 | N/A | N/A | 2 |
|  | *sgk-1(gf)* | 14.82±0.28 | 16 | 83/90 | 15* | < .0001* | 2 |
|  | *rict-1(mg451)* | 15.62±0.25 | 17 | 58/60 | N/A | N/A | 2 |
|  | *rict-1(mg451); sgk-1(gf)* | 11.84±0.20 | 13 | 58/60 | -24** | < .0001** | 2 |
|  | WT | 12.85±0.16 | 14 | 85/90 | N/A | N/A | 3 |
|  | *sgk-1(gf)* | 14.54±0.21 | 16 | 83/90 | 13* | < .0001* | 3 |
|  | *rict-1(mg451)* | 14.00±0.24 | 16 | 58/60 | N/A | N/A | 3 |
|  | *rict-1(mg451); sgk-1(gf)* | 11.76±0.21 | 12 | 59/60 | -16** | < .0001** | 3 |

Assay numbers indicate trials that were performed in parallel.

**Table S15. Lifespans shown in Figures 6F and 6G**

**20°C**

| E. coli | Strain | Mean lifespan (days±SEM) | 75^th^  percentile  (days) | No. animals | % Mean lifespan extension | *P* value against control | No. of Exp. | Figure |
| --- | --- | --- | --- | --- | --- | --- | --- | --- |
| OP50 bacteria | WT | 20.89±0.32 | 23 | 74/90 | N/A | N/A | 1 | 6F |
|  | *sgk-1(gf)* | 22.56±0.42 | 25 | 78/90 | 8* | < .0001* | 1 | 6F |
|  | *rict-1(mg451)* | 12.32±0.11 | 13 | 88/90 | N/A | N/A | 1 | 6F |
|  | *rict-1(mg451);*  *sgk-1(gf)* | 11.64±0.17 | 13 | 78/90 | -6** | .0190** | 1 | 6F |
| HT115  bacteria | WT | 21.83±0.22 | 23 | 75/90 | N/A | N/A | 1 | 6G |
|  | *sgk-1(gf)* | 24.74±0.52 | 29 | 80/90 | 13* | < .0001* | 1 | 6G |
|  | *rict-1(mg451)* | 15.54±0.26 | 17 | 87/90 | N/A | N/A | 1 | 6G |
|  | *rict-1(mg451);*  *sgk-1(gf)* | 14.60±0.14 | 15 | 89/90 | -6** | < .0004** | 1 | 6G |

(*) WT vs. *sgk-1*, (**) *rict-1* vs. *rict-1;sgk-1*

Animals from the parental generation were grown on OP50 or HT115. Synchronized young F1 adults were transferred to lifespan plates. *P* values were obtained by log-rank.

**Table S16. Lifespans shown in Figures 6H and 6I**

**20°C**

| Strain | RNAi treatment | RNAi mean lifespan (days±SEM) | 75^th^  percentile  (days) | No. RNAi animals | % Mean lifespan extension | *P* value against control | No. of Exp. | Figure |
| --- | --- | --- | --- | --- | --- | --- | --- | --- |
| WT | Control | 20.05±0.24 | 24 | 235/270 | N/A | N/A | 3 | 6H |
|  | *rict-1* | 24.46±0.28 | 26 | 240/270 | 22* | < .0001* | 3 | 6H |
|  | *sgk-1* | 19.15±0.21 | 22 | 259/270 | -4* | .002* | 3 | 6H |
| *sgk-1(gf)* | Control | 22.20±0.26 | 25 | 230/270 | N/A | N/A | 3 | 6I |
|  | *rict-1* | 24.73±0.24 | 27 | 237/270 | 11** | < .0001** | 3 | 6I |
|  | *sgk-1* | 23.55±0.25 | 26 | 223/270 | 6** | .0005** | 3 | 6I |

(*) WT control vs. *rict-1(RNAi)* or *sgk-1(RNAi)*, (**) *sgk-1* control vs. *sgk-1*;*rict-1(RNAi)* or *sgk-1*;*sgk-1(RNAi)*.

A composite of 3 individual experiments is tabulated, with individual trials described below. Animals from the parental generation were grown on HT115. Synchronized young F1 adults were transferred to lifespan plates. All treatments were performed during only adulthood, with pL4440 empty vector plates used for RNAi controls. *P* values were obtained by log-rank.

| Strain | RNAi treatment | RNAi mean lifespan (days±SEM) | 75^th^  percentile  (days) | No. RNAi animals | % Mean lifespan extension | *P* value against control | Assay  # |
| --- | --- | --- | --- | --- | --- | --- | --- |
| WT | Control | 20.09±0.45 | 24 | 78/90 | N/A | N/A | 1 |
|  | *rict-1* | 23.10±0.55 | 27 | 82/90 | 15* | < .0001* | 1 |
|  | *sgk-1* | 19.25±0.43 | 24 | 79/90 | -4* | .0807* | 1 |
| *sgk-1(gf)* | Control | 21.94±0.41 | 24 | 79/90 | N/A | N/A | 1 |
|  | *rict-1* | 24.48±0.38 | 26 | 81/90 | 12** | < .0001** | 1 |
|  | *sgk-1* | 23.05±0.40 | 26 | 75/90 | 5** | .0293** | 1 |
| WT | Control | 20.12±0.43 | 23 | 78/90 | N/A | N/A | 2 |
|  | *rict-1* | 21.93±0.43 | 25 | 84/90 | 9* | .0016* | 2 |
|  | *sgk-1* | 19.18±0.35 | 23 | 90/90 | -5* | .0251* | 2 |
| *sgk-1(gf)* | Control | 21.96±0.45 | 25 | 73/90 | N/A | N/A | 2 |
|  | *rict-1* | 24.23±0.47 | 27 | 78/90 | 10** | .0002** | 2 |
|  | *sgk-1* | 23.89±0.42 | 28 | 72/90 | 9** | .0055** | 2 |
| WT | Control | 19.95±0.39 | 23 | 79/90 | N/A | N/A | 3 |
|  | *rict-1* | 22.35±0.49 | 26 | 74/90 | 12* | < .0001* | 3 |
|  | *sgk-1* | 19.46±0.26 | 21 | 90/90 | -2* | .1161* | 3 |
| *sgk-1(gf)* | Control | 22.46±0.48 | 27 | 78/90 | N/A | N/A | 3 |
|  | *rict-1* | 25.49±0.40 | 27 | 78/90 | 13** | < .0001** | 3 |
|  | *sgk-1* | 23.71±0.45 | 28 | 76/90 | 6** | .0506** | 3 |

Assay numbers indicate trials that were performed in parallel.

**Table S17. Lifespans shown in Figure 6J**

**20°C**

| Strain | RNAi treatment | RNAi mean lifespan (days±SEM) | 75^th^  percentile  (days) | No. RNAi animals | % Mean lifespan extension | *P* value against control | No. of Exp. | Figure |
| --- | --- | --- | --- | --- | --- | --- | --- | --- |
| *sid-1* | Control | 20.15±0.35 | 22 | 87/90 | N/A | N/A | 1 | 6J |
| *(neuronal RNAi)* | *rict-1* | 17.41±0.40 | 20 | 86/90 | -14 | .0001 | 1 | 6J |

Animals from the parental generation were grown on HT115. Synchronized young F1 adults were transferred to lifespan plates. All treatments were performed during only adulthood, with pL4440 empty vector plates used for RNAi controls. *P* values were obtained by log-rank.

**Table S18. Lifespans shown in supplementary Figure 1**

**25°C**

| Strain | RNAi treatment | RNAi mean life span (days±SEM) | 75^th^  percentile  (days) | No. RNAi animals | % Mean life span extension | *P* value against control | No. of Exp. | Suppl. Figure |
| --- | --- | --- | --- | --- | --- | --- | --- | --- |
| *glp-1(bn18)* | Control | 15.77±0.22 | 17 | 86/90 | N/A | N/A | 1 | 1 |
|  | *rict-1* | 17.69±0.26 | 19 | 87/90 | 12 | .0001 | 1 | 1 |

Animals from the parental generation were grown on HT115. Synchronized young F1 adults were transferred to lifespan plates. All treatments were performed during only adulthood, with pL4440 empty vector plates used for RNAi controls. *P* values were obtained by log-rank.

**Table S19. Lifespans shown in supplementary Figure 3**

**25°C**

| Strain | RNAi treatment | RNAi mean life span (days±SEM) | 75^th^  percentile  (days) | No. RNAi animals | % Mean life span extension | *P* value against control | No. of Exp. | Suppl. Figure |
| --- | --- | --- | --- | --- | --- | --- | --- | --- |
| WT | Control | 11.42±0.34 | 14 | 72/90 | N/A | N/A | 1 | 3A |
|  | *rict-1* | 13.91±0.42 | 16 | 66/90 | 22* | < .0001* | 1 | 3A |
|  | *sgk-1* | 12.82±0.37 | 15 | 84/90 | 12* | .0049* | 1 | 3A |
| *daf-16(mgDf47)* | Control | 10.80±0.30 | 13 | 80/90 | N/A | N/A | 1 | 3B |
|  | *rict-1* | 12.35±0.31 | 14 | 75/90 | 14** | .0003** | 1 | 3B |
|  | *sgk-1* | 12.52±0.30 | 14 | 81/90 | 16** | .0004** | 1 | 3B |

(*) WT control vs. *rict-1(RNAi)* or *sgk-1(RNAi)*, (**) *daf-16* control vs. *daf-16;rict-1(RNAi)* or *daf-16;sgk-1(RNAi)*.

Animals from the parental generation were grown on HT115. Synchronized young F1 adults were transferred to lifespan plates. All treatments were performed during only adulthood, with pL4440 empty vector plates used for RNAi controls. *P* values were obtained by log-rank.

**Table S20. Lifespans shown in supplementary Figure 7**

**25°C**

| Strain | medium | Mean life span (days±SEM) | 75^th^  percentile  (days) | No. RNAi animals | % Mean life span extension | *P* value against control | No. of Exp. | Suppl. Figure |
| --- | --- | --- | --- | --- | --- | --- | --- | --- |
| WT | OP50 | 11.38±0.18 | 12 | 77/90 | N/A | N/A | 1 | 7A |
|  | OP50+L-Trp | 8.44±0.14 | 9 | 78/90 | -26 | < .0001 | 1 | 7A |
| *rict-1(mg451)* | OP50 | 8.26±0.14 | 9 | 73/90 | N/A | N/A | 1 | 7B |
|  | OP50+L-Trp | 7.46±0.13 | 8 | 72/90 | -10 | < .0001 | 1 | 7B |

Animals from the parental generation were grown on OP50. Synchronized young F1 adults were transferred to lifespan plates that did or did not contain L-tryptophan (L-Trp). *P* values were obtained by log-rank.

**Table S21. Summary of lifespans at 25°C**

| E. coli | Strain | Mean lifespan (days±SEM) | 75^th^  percentile  (days) | No. animals | % Mean lifespan extension | *P* value | *P* value compared to | No. of Exp. | Figure |
| --- | --- | --- | --- | --- | --- | --- | --- | --- | --- |
| OP50 bacteria | WT | 11.61±0.07 | 13 | 334/340 | N/A | N/A | N/A | 3 | 2A |
|  | *rict-1(mg451)* | 8.87±0.11 | 10 | 330/336 | -24 | < .0001 | WT | 3 | 2A |
|  | *skn-1(zu135)* | 10.46±0.17 | 12 | 158/170 | N/A | N/A | N/A | 3 | 2A |
|  | *rict-1(mg451);*  *skn-1(zu135)* | 9.38±0.19 | 12 | 180/190 | -10 | < .0009 | *skn-1*  *(zu135)* | 3 | 2A |
|  | WT | 12.19±0.14 | 13 | 175/180 | N/A | N/A | N/A | 3 | 5D |
|  | *rict-1(mg451)* | 8.88±0.14 | 10 | 178/180 | -27 | < .0001 | WT | 3 | 5D |
|  | *sgk-1(ok538)* | 9.66±0.14 | 11 | 171/180 | -18 | < .0001 | WT | 3 | 5D |
|  | WT | 12.91±0.13 | 14 | 239/270 | N/A | N/A | N/A | 3 | 6D |
|  | *sgk-1(gf)* | 12.30±0.14 | 14 | 225/270 | -5 | .0065 | WT | 3 | 6D |
|  | *rict-1(mg451)* | 10.49±0.08 | 11 | 266/270 | N/A | N/A | N/A | 3 | 6D |
|  | *rict-1(mg451);*  *sgk-1(gf)* | 9.86±0.05 | 10 | 232/270 | -6 | < .0001 | *rict-1*  *(mg451)* | 3 | 6D |
| HT115  bacteria | WT | 12.83±0.11 | 14 | 324/330 | N/A | N/A | N/A | 3 | 2B |
|  | *rict-1(mg451)* | 14.15±0.14 | 16 | 319/330 | 10 | < .0001 | WT | 3 | 2B |
|  | *skn-1(zu135)* | 11.97±0.16 | 13 | 140/140 | N/A | N/A | N/A | 3 | 2B |
|  | *rict-1(mg451);*  *skn-1(zu135)* | 10.78±0.18 | 13 | 156/162 | -10 | < .0001 | *skn-1*  *(zu135)* | 3 | 2B |
|  | WT | 13.99±0.15 | 15 | 165/180 | N/A | N/A | N/A | 3 | 5E |
|  | *rict-1(mg451)* | 14.64±0.17 | 16 | 163/180 | 5 | < .0001 | WT | 3 | 5E |
|  | *sgk-1(ok538)* | 15.45±0.23 | 18 | 177/180 | 10 | < .0001 | WT | 3 | 5E |
|  | WT | 13.02±0.12 | 14 | 255/270 | N/A | N/A | N/A | 3 | 6E |
|  | *sgk-1(gf)* | 14.58±0.12 | 16 | 250/270 | 12 | < .0001 | WT | 3 | 6E |
|  | *rict-1(mg451)* | 14.45±0.15 | 16 | 195/210 | N/A | N/A | N/A | 3 | 6E |
|  | *rict-1(mg451);*  *sgk-1(gf)* | 11.44±0.13 | 13 | 196/210 | -21 | < .0001 | *rict-1*  *(mg451)* | 3 | 6E |

This table summarizes lifespan data shown in the indicated main figures.

| Strain | RNAi treatment | RNAi mean lifespan (days±SEM) | 75^th^  percentile  (days) | No. RNAi animals | % Mean lifespan extension | *P* value compared to Control | No. of Exp. | Figure |
| --- | --- | --- | --- | --- | --- | --- | --- | --- |
| WT | Control | 11.83±0.15 | 13 | 201/210 | N/A | N/A | 3 | 5A |
|  | *rict-1* | 14.02±0.15 | 15 | 210/212 | 19 | < .0001 | 3 | 5A |
|  | *sgk-1* | 13.12±0.21 | 15 | 189/210 | 11 | < .0001 | 3 | 5A |
| *skn-1*  *(zu135)* | Control | 10.28±0.15 | 11 | 118/130 | N/A | N/A | 3 | 5B |
|  | *rict-1* | 10.65±0.17 | 12 | 124/131 | 4 | .0314 | 3 | 5B |
|  | *sgk-1* | 10.84±0.18 | 12 | 111/120 | 5 | .0066 | 3 | 5B |
| *daf-16 (mgDf47)* | Control | 9.06±0.09 | 10 | 250/270 | N/A | N/A | 3 | 5C |
|  | *rict-1* | 10.66±0.13 | 12 | 254/270 | 18 | < .0001 | 3 | 5C |
|  | *sgk-1* | 10.29±0.13 | 11 | 236/270 | 14 | < .0001 | 3 | 5C |
| WT | Control | 11.63±0.15 | 13 | 243/270 | N/A | N/A | 3 | 6B |
|  | *rict-1* | 13.45±0.16 | 15 | 244/270 | 16 | < .0001 | 3 | 6B |
| *sgk-1(gf)* | Control | 12.91±0.12 | 14 | 244/270 | N/A | N/A | 3 | 6C |
|  | *rict-1* | 12.40±0.13 | 14 | 241/270 | -4 | .0232 | 3 | 6C |

This table summarizes lifespan data shown in the indicated main figures.

**Table S22. Summary of lifespans at 20°C**

| E. coli | Strain | Mean lifespan (days±SEM) | 75^th^  percentile  (days) | No. animals | % Mean lifespan extension | *P* value | *P* value compared to | No. of Exp. | Figure |
| --- | --- | --- | --- | --- | --- | --- | --- | --- | --- |
| OP50 bacteria | WT | 20.68±0.18 | 22 | 183/270 | N/A | N/A | N/A | 3 | 2C |
|  | *rict-1(mg451)* | 12.52±0.10 | 13 | 213/270 | -40 | < .0001 | WT | 3 | 2C |
|  | *sgk-1(ok538)* | 13.46±0.17 | 15 | 220/270 | -35 | < .0001 | WT | 3 | 2C |
|  | WT | 20.89±0.32 | 23 | 74/90 | N/A | N/A | N/A | 1 | 6F |
|  | *sgk-1(gf)* | 22.56±0.42 | 25 | 78/90 | 8 | < .0001 | WT | 1 | 6F |
|  | *rict-1(mg451)* | 12.32±0.11 | 13 | 88/90 | N/A | N/A | N/A | 1 | 6F |
|  | *rict-1(mg451);*  *sgk-1(gf)* | 11.64±0.17 | 13 | 78/90 | -6 | .0190 | *rict-1*  *(mg451)* | 1 | 6F |
| HT115  bacteria | WT | 21.45±0.16 | 23 | 154/270 | N/A | N/A | N/A | 3 | 2D |
|  | *rict-1(mg451)* | 15.78±0.19 | 17 | 221/270 | -26 | < .0001 | WT | 3 | 2D |
|  | *sgk-1(ok538)* | 16.43±0.16 | 18 | 245/270 | -23 | < .0001 | WT | 3 | 2D |
|  | WT | 21.83±0.22 | 23 | 75/90 | N/A | N/A | N/A | 1 | 6G |
|  | *sgk-1(gf)* | 24.74±0.52 | 29 | 80/90 | 13 | < .0001 | WT | 1 | 6G |
|  | *rict-1(mg451)* | 15.54±0.26 | 17 | 87/90 | N/A | N/A | N/A | 1 | 6G |
|  | *rict-1(mg451);*  *sgk-1(gf)* | 14.60±0.14 | 15 | 89/90 | -6 | < .0004 | *rict-1*  *(mg451)* | 1 | 6G |

This table summarizes lifespan data shown in the indicated main figures.

| Strain | RNAi treatment | RNAi mean lifespan (days±SEM) | 75^th^  percentile  (days) | No. RNAi animals | % Mean lifespan extension | *P* value compared to Control | No. of Exp. | Figure |
| --- | --- | --- | --- | --- | --- | --- | --- | --- |
| WT | Control | 20.05±0.24 | 24 | 235/270 | N/A | N/A | 3 | 6H |
|  | *rict-1* | 24.46±0.28 | 26 | 240/270 | 22 | < .0001 | 3 | 6H |
|  | *sgk-1* | 19.15±0.21 | 22 | 259/270 | -4 | .002 | 3 | 6H |
| *sgk-1(gf)* | Control | 22.20±0.26 | 25 | 230/270 | N/A | N/A | 3 | 6J |
|  | *rict-1* | 24.73±0.24 | 27 | 237/270 | 11 | < .0001 | 3 | 6I |
|  | *sgk-1* | 23.55±0.25 | 26 | 223/270 | 6 | .0005 | 3 | 6I |
| *sid-1(neuronal RNAi strain)* | Control | 20.15±0.35 | 22 | 87/90 | N/A | N/A | 1 | 6J |
|  | *rict-1* | 17.41±0.40 | 20 | 86/90 | -14 | .0001 | 1 | 6J |

This table summarizes lifespan data shown in the indicated main figures.

**Table S23. Nematode strains used in this study**

| Number | Genetic background | Transgene | Array number | Referenced |
| --- | --- | --- | --- | --- |
| LD001 | N2 | Is[*SKN-1B/C::GFP*] | 007 | An & Blackwell, 2003 |
| ENH196 | *sgk-1(ok538)* | Is[*SKN-1B/C::GFP*] | 007 | This study |
| LD1546 | *rict-1(mg451)* | Is[*SKN-1B/C::GFP*] | 007 | This study |
| LD1171 | N2 | Is[*gcs-1p::GFP*] | 003 | Wang *et al*., 2010 |
|  | *rict-1(mg451)* | Is[*gcs-1p::GFP*] | 003 | This study |
|  | N2 | [*gst-4p::GFP*] |  | Link & Johnson, 2002 |
| LD1175 | *skn-1(zu67)* | Is[*gcs-1p::GFP*] | 003 | Wang *et al*., 2010 |
| ENH353 | N2 | Is[*SGK-1::GFP*] |  | Hertweck *et al*., 2004 |
| ENH359 | *rict-1(mg451)* | Is[*SGK-1::GFP*] |  | This study |
| EU1 | *skn-1(zu67)* |  |  | Bowerman *et al*., 1992 |
| EU31 | *skn-1(zu135)* |  |  | Bowerman *et al*., 1992 |
|  | *daf-16(mgDf47)* |  |  | Ogg *et al*., 1997 |
| BR4774 | *sgk-1(ok538)* |  |  | Hertweck *et al*., 2004 |
| ENH235 | *sgk-1(ft15)* |  |  | Jones *et al*., 2009 |
| ENH190 | *rict-1(mg451)* |  |  | Soukas *et al*., 2009 |
| ENH177 | *rict-1(mg451);skn-1(zu67)* |  |  | This study |
| LD1461 | *rict-1(mg451);skn-1(zu135)* |  |  | This study |
| ENH337 | *rict-1(mg451);sgk-1(ft15)* |  |  | This study |
| TU3595 | uIs72 [pCFJ90 (P_myo-2_mCherry), P_unc-119_sid-1, P_mec-18_mec-18::gfp]; sid-1(pk3321) him-5(e1490); lin-15b(n744) |  |  | Calixto et al., 2010 |
| DG2389 | *glp-1(bn18)* |  |  | Kodoyianni et al., 1992 |

Strains *rict-1(mg451)* and *sgk-1(ft15)* that were obtained from external sources were outcrossed with WT at least 4 times. ENH235 is derived from the *sgk-1(ft15)* strain KQ1564, in which an *akt-2* deletion present in the original isolate has been removed. This was confirmed by genotyping.
