# Supplementary material for: mTORC2-SGK-1 acts in two environmentally responsive pathways with opposing effects on longevity
Source: Aging Cell. 2014 Jul 9;13(5):869–78. doi: 10.1111/acel.12248 (PMC4172656; doi:10.1111/acel.12248)
Supplement: Supplementary file 9 — Appendix S1. Experimental procedures. [file acel0013-0869-sd9.docx]

**Supporting Information**

**Experimental Procedures**

**Strains**

Experimental animals were maintained on solid Nematode Growth Medium (NGM). Experiments were performed on animals maintained at 20°C except where stated otherwise. Nematode strains used in this study are described in Table S23.

Strains obtained from external sources were outcrossed at least 4 times.

**RNAi treatment**

Feeding RNAi was performed as described, using HT115 and the pL4440 vector as control (Wang *et al*., 2010). RNAi constructs were taken from the ORFeome RNAi library (Rual *et al.*, 2004) and confirmed by sequencing. For lifespan experiments that required feeding RNAi, animals were exposed to RNAi bacteria starting from the beginning of adulthood.

**L-Tryptophan supplementation**

L-Tryptophan (Kanto Chemical) was dissolved in water at 12.5 mg/ml, and 100 μl of this solution was placed on top of the OP50 lawn as described (Gracida & Eckmann, 2013).

**Transgenic reporter scoring**

Expression level of transgenic GFP reporters and nuclear accumulation of SKN-1::GFP were scored as “Low, Medium, or High” as published (An & Blackwell, 2003; Tullet *et al*., 2008; Wang *et al*., 2010; Robida-Stubbs *et al*., 2012). For promoter reporters, “High” indicates that GFP signal was detected at high levels throughout most of the intestine, while “Medium” refers to animals in which robust GFP signal was present only anteriorly or posteriorly. For the SKN-1::GFP fusion reporters, High indicated that a strong SKN-1::GFP signal was present in all intestinal nuclei, and Medium that nuclear SKN-1::GFP was present at high levels anteriorly, posteriorly or both, but barely visible midway through the intestine, or that a weak signal was observed in all intestinal nuclei. In RNAi or OP50 vs HT115 feeding assays, L4 stage animals were placed on plates and allowed to lay eggs, and progeny were allowed to develop to the L4 or early adult stage prior to scoring. In L-Tryptophan supplementation assays L4 animals were placed on OP50 plates containing L-Tryptophan, and their progenies were scored at the L4 or early adult stage. *P* values were determined from a chi^2^ test.

**Oxidative stress assays**

To assay t-butyl hydrogen peroxide (TBHP) resistance, animals were fed with OP50 or HT115 for two generations, then young adults were transferred to M9 solution that contained 6 mM TBHP (Sigma-Aldrich) and the respective bacterial food. In Arsenite (As) resistance assays, animals were fed with OP50 or HT115 for two generations, then young adults were transferred to M9 solution that contained 2.5 mM NaAsO_2_ (Sigma-Aldrich) and the respective bacterial food. In RNAi experiments, L4 stage animals were placed on RNAi plates and allowed to lay eggs. Young F1 adults were transferred to M9 containing 2.5 mM NaAsO_2_ and the respective bacterial food, then periodically scored for survival. All data were analyzed using JMP. P values were calculated by log-rank.

**Lifespan assays**

All lifespans were measured from the beginning of adulthood. To determine mean lifespan at 15°C, 20°C or 25°C, animals from the parental generation were raised and maintained at 20°C on the same food source that would be used for lifespan analysis. After synchronization by timed egg lay for 6 hours, young adults were transferred to lifespan plates at the same temperature containing 5-fluoro-2′-deoxyuridine (FUdR) (100 μg/ml) to prevent progeny development. For lifespan assays of *glp-1* animals, animals were synchronized on HT115 plates at 20°C. Plates were placed at 25°C within 6 hours of hatching to cause sterility, then young adults were transferred to RNAi lifespan plates containing FUdR at 25°C. RNAi treatments were performed only during adulthood. Synchronized young adults were transferred to life span plates seeded with gene-specific or control RNAi bacteria. L-Tryptophan treatments were performed only during adulthood, with FUdR present (100 μg/ml) to prevent progeny development. The first day of adulthood was used as t=0, with animals scored each day. Those that crawled off the plate, exploded, or bagged were censored. JMP version 9, was used for statistical analyses, and *P* values were calculated using the log-rank method.

**DR and liquid culture experiments**

Liquid dietary restriction was performed by a modification of a published protocol (Mair *et al*., 2009) that consistently increased WT lifespan and stress resistance (N. M. and T.K.B., unpublished data). Bacterial growth was arrested using ampicillin, tetracycline, and kanamycin. In all experiments, worms were placed in liquid culture on day 3 of adulthood, and these cultures were constantly rotated to distribute the food and enhance oxygenation. Survival plots and *P* values (Log-Rank) were determined with JMP 8.0.2 software.

**In vitro protein kinase assay**

ENH353 *Is[SGK-1::GFP]* and ENH359 *rict-1(mg451);Is[SGK-1::GFP]* were fed with OP50, HT115, and OP50 supplemented with L-Tryptophan, respectively. Purification of tagged-proteins and in vitro kinase assays were performed as described (Hertweck et al., 2004; Tullet et al., 2008). In brief, SGK-1 fused to GFP was isolated from sonicated worm lysates of WT and *rict-1(mg451)* mutants, using a monoclonal GFP antibody (Santa Cruz, sc-9996). Purified SGK-1::GFP and bacterially expressed GST-SKN-1C fusion protein were then incubated in kinase buffer (10mM HEPES pH 7.4; 5mM DTT; 10mM MgCl_2_ , 25um ATP) containing [γ-^32^P] ATP at 25°C for 5 min. Samples were analyzed by SDS-PAGE and detected by autoradiography.

**Food avoidance assays**

A microbial avoidance assay was performed essentially as published (Melo and Ruvkun, 2012). To assay food avoidance on OP50 or HT115, animals from the parental generation were raised and maintained at 20°C on the same food source that would be used for the assay. Animals were then synchronized by timed egg lay for 6 hours on plates at 20°C. Synchronized day-1 adults were transferred to either OP50 or HT115 assay plate, then numbers of animals on or off the food were counted after 3, 6, and 24 hours. Results were derived from 3 replicates of 30 animals. Error bars indicate the mean ± SEM. *P* values were determined by *t*-test.

**Supplementary Figure Legends**

**Supplementary Figure 1. *rict-1* RNAi increases lifespan in *glp-1(bn18)* mutants.** *rict-1* RNAi increases lifespan in *glp-1(bn18)* mutants, in which germ cell proliferation is blocked at the non-permissive temperature of 25°C (Kenyon, 2010). Animals were placed at 25°C from hatching until adulthood, then RNAi was initiated. Statistics are provided in Table S18.

**Supplementary Figure 2. *rict-1* or *sgk-1* knockdown activates SKN-1 target genes.**

(A-C) *skn-1*-dependent activation of the SKN-1 target promoters *gcs-1* and *gst-4*. All analyses were performed at 20°C. For each comparison to control, P values were derived from a chi^2^ test, ***P<0.0001; NS, not significant. **(**D) *sgk-1* mutation increased intestinal SKN-1 nuclear occupancy in animals that were fed HT115. SKN-1B/C::GFP encodes two of three SKN-1 isoforms. ***P<0.0001; NS, not significant.

**Supplementary Figure 3. FUdR does not affect lifespan extension from *rict-1* or *sgk-1* RNAi.**

**(A, B)** Lifespan was determined for WT or *daf-16(mgDf47)* animals in the absence of FUdR. RNAi treatments were performed only during adulthood at 25°C. Mean lifespan from day-one adulthood is shown in parentheses. Statistics are provided in Table S19.

**Supplementary Figure 4. Full-length gels corresponding to Figure 4F.**

(A) Phosphorylated GST-SKN-1C was separated by SDS-PAGE and detected by autoradiography. (B) GST-SKN-1C proteins were stained with Coomassie Brilliant Blue. (C) Purified SGK-1::GFP from cell extracts were separated by SDS-PAGE and detected by immunoblotting with anti-GFP.

**Supplementary Figure 5. *rict-1* mutants avoid OP50 and HT115 similarly.**

Synchronized day-1 adults were transferred to either OP50 or HT115 assay plates, then numbers of animals on and off the food were scored after 3 (A), 6 (B), or 24 (C) hours. Error bars indicate the mean ± SEM. *P* values were determined by *t*-test.

**Supplementary Figure 6. L-Tryptophan supplementation** **induces SKN-1 target genes.**

(A-D) L-Tryptophan (L-Trp) supplementation under conditions that rescue the *nhr-114* reproductive defect (Gracida and Eckmann, 2013) increased intestinal SKN-1 nuclear occupancy, and activated the SKN-1 target promoter *gcs-1*. OP50-seeded plates were supplemented with L-Trp (Materials and Methods) prior to assay. In (C) and (D) the *rict-1(mg451)* allele was analyzed. All assays were performed at 20^o^C. For each comparison to control, P values were derived from a chi^2^ test, ***P<0.0001; **P<0.001; *P<0.01; NS, not significant.

**Supplementary Figure 7. L-Tryptophan supplementation decreases WT lifespan**. OP50-seeded plates were supplemented with L-Trp as in Supplementary Figure 6. Mean lifespans are shown in parentheses. Statistics are provided in Table S20. WT lifespan was also decreased by administration of L-Trp at a 2-fold lower concentration (not shown).

**Additional citations**

Wang J, Robida-Stubbs S, Tullet JM, Rual JF, Vidal M, Blackwell TK (2010). RNAi screening implicates a SKN-1-dependent transcriptional response in stress resistance and longevity deriving from translation inhibition. *PLoS Genet*. **6,** e1001048.

Rual JF, Ceron J, Koreth J, Hao T, Nicot AS, Hirozane-Kishikawa T, Vandenhaute J, Orkin SH, Hill DE, van den Heuvel S, Vidal M (2004). Toward improving Caenorhabditis elegans phenome mapping with an ORFeome-based RNAi library. *Genome Res*. **14**, 2162-2168.

[Kodoyianni, V.](http://www.wormbase.org/resources/person/WBPerson1176), [Maine, E. M.](http://www.wormbase.org/resources/person/WBPerson398), & [Kimble, J. E.](http://www.wormbase.org/resources/person/WBPerson320) (1992). Molecular basis of loss-of-function mutations in the glp-1 gene of Caenorhabditis elegans. *Mol Biol Cell, 3*, 1199-213. Mair W, Panowski SH, Shaw RJ, Dillin A (2009). Optimizing dietary restriction for genetic epistasis analysis and gene discovery in C. elegans. *PLoS One*. **4**, e4535.

Link CD, Johnson CJ (2002). Reporter transgenes for study of oxidant stress in Caenorhabditis elegans. *Methods Enzymol*. **353**, 497-505.

Ogg S, Paradis S, Gottlieb S, Patterson GI, Lee L, Tissenbaum HA, Ruvkun G (1997). The Fork head transcription factor DAF-16 transduces insulin-like metabolic and longevity signals in C. elegans. *Nature*. **389**, 994-999.

Kodoyianni V, Maine EM, Kimble JE (1992). Molecular basis of loss-of-function mutations in the glp-1 gene of Caenorhabditis elegans. *Mol Biol Cell*. **3**, 1199-1213.
